# Casein kinase 2 phosphorylates and induces the SALL2 tumor suppressor degradation in colon cancer cells

**DOI:** 10.1038/s41419-024-06591-z

**Published:** 2024-03-16

**Authors:** V. E. Hermosilla, L. Gyenis, A. J. Rabalski, M. E. Armijo, P. Sepúlveda, F. Duprat, D. Benítez-Riquelme, F. Fuentes-Villalobos, A. Quiroz, M. I. Hepp, C. Farkas, M. Mastel, I. González-Chavarría, R. Jackstadt, D. W. Litchfield, A. F. Castro, R. Pincheira

**Affiliations:** 1 Departamento de Bioquímica y Biología Molecular, Facultad de Ciencias Biológicas, Universidad de Concepción, Concepción, Chile; 2 Laboratorio de Transducción de Señales y Cáncer, Facultad de Ciencias Biológicas, Universidad de Concepción, Concepción, Chile; 3 Department of Biochemistry, Schulich School of Medicine & Dentistry, University of Western Ontario, London, ON Canada; 4 Departamento de Fisiopatología, Facultad de Ciencias Biológicas, Universidad de Concepción, Concepción, Chile; 5 Heidelberg Institute for Stem Cell Technology and Experimental Medicine (HI-STEM gGmbH), 69120 Heidelberg. Cancer Progression and Metastasis Group, German Cancer Research Center (DKFZ) and DKFZ-ZMBH Alliance, 69120 Heidelberg, Germany; 6 Faculty of Biosciences, Heidelberg University, 69120 Heidelberg, Germany; 7 Present Address: Dept of Orofacial Sciences and Dept of Anatomy, University of California-San Francisco, San Francisco, CA USA; 8 Present Address: Odyssey Therapeutics, Boston, MA USA; 9 Present Address: Laboratorio de Inmunovirología. Departamento de Microbiologia. Facultad de Ciencias Biológicas, Universidad de Concepción, Concepción, Chile; 10 Present Address: Laboratorio de Investigación en Ciencias Biomédicas, Departamento de Ciencias Básicas y Morfología, Facultad de Medicina, Universidad Católica de la Santísima Concepción, Concepción, Chile

**Keywords:** Molecular biology, Biochemistry

## Abstract

Spalt-like proteins are Zinc finger transcription factors from *Caenorhabditis elegans* to vertebrates, with critical roles in development. In vertebrates, four paralogues have been identified (SALL1-4), and SALL2 is the family’s most dissimilar member. SALL2 is required during brain and eye development. It is downregulated in cancer and acts as a tumor suppressor, promoting cell cycle arrest and cell death. Despite its critical functions, information about SALL2 regulation is scarce. Public data indicate that SALL2 is ubiquitinated and phosphorylated in several residues along the protein, but the mechanisms, biological consequences, and enzymes responsible for these modifications remain unknown. Bioinformatic analyses identified several putative phosphorylation sites for Casein Kinase II (CK2) located within a highly conserved C-terminal PEST degradation motif of SALL2. CK2 is a serine/threonine kinase that promotes cell proliferation and survival and is often hyperactivated in cancer. We demonstrated that CK2 phosphorylates SALL2 residues S763, T778, S802, and S806 and promotes SALL2 degradation by the proteasome. Accordingly, pharmacological inhibition of CK2 with Silmitasertib (CX-4945) restored endogenous SALL2 protein levels in *SALL2*-deficient breast MDA-MB-231, lung H1299, and colon SW480 cancer cells. Silmitasertib induced a methuosis-like phenotype and cell death in SW480 cells. However, the phenotype was significantly attenuated in CRISPr/Cas9-mediated *SALL2* knockout SW480 cells. Similarly, *Sall2*-deficient tumor organoids were more resistant to Silmitasertib-induced cell death, confirming that SALL2 sensitizes cancer cells to CK2 inhibition. We identified a novel CK2-dependent mechanism for SALL2 regulation and provided new insights into the interplay between these two proteins and their role in cell survival and proliferation.

## Introduction

SPALT-like proteins are multi-Zinc finger transcription factors from *Caenorhabditis elegans* to vertebrates, with pivotal roles in development [1]. Four paralogues -named Sall1 to 4- have been identified in vertebrates [1]. In agreement with their developmental role, mutations in *SALL1*, *SALL3*, and *SALL4* are involved in human syndromes [2–6]. However, the role of SALL2 is less clear. SALL2 is the most dissimilar member among vertebrate SPALT-like proteins, with C-terminal Zn fingers that differ from those found in the other members of the family and with distinct DNA-binding properties [7]. The differential region of SALL2 corresponds to the transactivation domain [8] and the binding site for the oncogenic polyomavirus large T antigen required for viral replication [9].

*SALL2/Sall2* is involved in the proper closure of the neural tube and the optic fissure [10, 11], morphological differentiation of hippocampal neurons [12], stemness maintenance of conjunctival epithelial stem cells [13], inhibition of DNA synthesis and entry into quiescence [14], promotion of cell death under genotoxic stimuli [15–17], and cell migration of cancer cells and embryonic fibroblasts [7–19]. SALL2 binds to the cognate sequence GGG(T/C)GGG in promoters of target genes [15]. Specifically, it binds and activates promoters of cell cycle inhibitors p21 (*CDKN1A*) and p16 (*CDKN2A*), proapoptotic genes such as *BAX* and NOXA (*PMAIP1*) [8, 15, 16, 20], and Integrin β1 (*ITGβ1)* [19], a membrane receptor involved in cell adhesion and a variety of processes including embryogenesis, tissue repair, and cancer progression. SALL2 also represses promoters of the oncogenes MYC (*c-MYC)*, Cyclin D1 (*CCND1*), and Cyclin E1 (*CCNE1*) [21, 22]. *SALL2* is downregulated in different types of cancer, such as leukemia, lung, prostate, colon, and ovarian cancer [23–26]. Ectopic expression of SALL2 diminished the tumorigenic potential of ovarian cancer cells when inoculated into SCID mice [8]. Accordingly, *SALL2* has been proposed as a tumor suppressor. However, SALL2’s role in cancer is controversial because it is upregulated in Wilms’ tumors, synovial sarcoma, testicular germ cell tumors, oral tongue squamous cell carcinoma, and glioblastoma [26, 27]. Therefore, it is crucial to identify regulatory mechanisms for *SALL2* expression to understand further how this gene is deregulated under pathological settings. Promoter methylation, histone acetylation, and loss of heterozygosity account for epigenetic mechanisms and chromosomal events underlying the downregulation of *SALL2* in cancer [18, 24, 28–31].

Posttranslational modifications (PTMs) constitute a rapid and reversible means to regulate the activity and availability of transcription factors [32]; phosphorylation is the most common -yet specific- PTM [33]. Massive proteomic analyses showed that SALL2 is widely phosphorylated along its sequence [34]. However, there is no information about the enzymes involved, the cellular context, or the molecular and cellular effects of SALL2 phosphorylation. We followed a bioinformatic approach to identify possible mechanisms for SALL2 posttranslational regulation, identifying four putative phosphorylation sites matching the S/T-X-X-E/D, CK2 consensus sequence [35]. These sites were clustered next to the DNA-binding domain of SALL2 in a potential PEST (proline, aspartic acid, serine, threonine) degradation motif [36].

CK2 is a constitutively active serine/threonine kinase whose pleiotropic activity involves epithelial-to-mesenchymal transition, cell survival, proliferation, chemoresistance, and angiogenesis, among other functions [37–39]. This ubiquitous and highly conserved kinase exhibits a heterotetrameric structure composed of two regulatory β subunits and two catalytic α and/or α’ subunits [40]. More than 300 CK2 substrates have been identified [41, 42]. CK2-dependent phosphorylation impacts substrate subcellular localization, activity, stability, or susceptibility to caspase cleavage [43–47]. In cancer, CK2 activity and expression are often elevated, exerting an oncogenic role through the upregulation of oncogenes and downregulation of tumor suppressors [37, 40]. In agreement with its oncogenic function, CK2 inhibition has been related to cell cycle arrest, apoptosis, autophagic cell death, and, most recently, to methuosis-like cell death [42–45]. Methuosis is a non-apoptotic form of cell death characterized by a Ras-dependent cytoplasmic accumulation of large fluid-filled vacuoles derived from macropinosomes [48].

Here, we identified SALL2 as a novel CK2 substrate. We found that CK2 triggers SALL2 ubiquitylation and its proteasome-dependent degradation. Our data described the first phosphorylation-dependent regulation of SALL2, adding new insights into the molecular mechanisms by which this tumor suppressor is downregulated in cancer cells. Importantly, our data suggest that CK2 inhibition with Silmitasertib, formerly CX-4945, triggers cell death in a SALL2-dependent manner. Because Silmitarsertib is currently in clinical trials for cholangiocarcinoma, medulloblastoma, myeloma, basal cell carcinoma, and advanced solid tumors (Clinical trials.gov ID: NCT03904862, NCT01199718, NCT03897036, NCT00891280), molecular pathways involved in Silmitasertib cytotoxicity are of great interest to ensure a successful treatment.

## Results

### SALL2 is a novel CK2 substrate in vitro

**Fig. 1:**
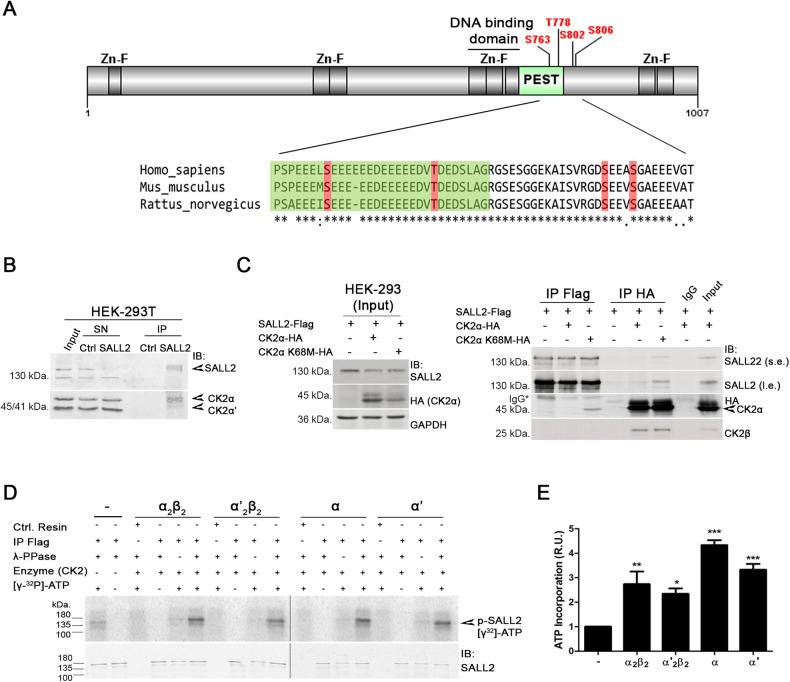
SALL2 protein is a CK2 substrate in vitro. **A** Scheme of human SALL2 protein. Zinc Finger motifs are highlighted in dark gray, and a putative C-terminal PEST motif is shown in green next to the DNA-binding domain. Putative phosphorylation sites matching the CK2 consensus sequence are indicated in red. Alignment of protein sequences from *Homo sapiens* (NP_005398.2), *Mus musculus* (NP_056587.2), and *Rattus Norvegicus* (NP_001100732.1) with Clustal omega [92] indicated that this region is highly conserved. **B** Endogenous SALL2 and CK2α interact in HEK 293T cells. Immunoprecipitations (IP) were performed using anti-SALL2 antibody (Bethyl Lab.), SN supernatant. **C** SALL2-Flag protein was co-expressed with HA-tagged wild-type CK2α or the K68M dominant-negative mutant. Immunoprecipitation assays were performed using Flag or HA antibodies. s.e., short exposure; l.e., long exposure. **D** SALL2-Flag was overexpressed in Flp-In™ T-REx™ U2OS cells, followed by immunoprecipitation for in vitro phosphorylation reactions. Different forms of recombinant CK2 were used, including the CK2αβ and CK2α’β holo forms and the CK2α and CK2α’ monomeric subunits, all of which phosphorylate SALL2 in vitro. Ctrl. Resin, immunoprecipitation without antibody; λ-PPase, dephosphorylation with λ-phosphatase prior to the in vitro kinase reaction. **E** Quantification of (**D**). R.U. relative units to the basal condition; error bars equal SEM; *p < 0.05, **p < 0.01, ***p < 0.001, n.s. non-significant *p*-value (one-way ANOVA plus Tukey’s multiple comparison test).

Previous proteomic studies have shown that SALL2 is phosphorylated at specific sites along its entire sequence [34]. However, there is no further information about the kinases involved or the occurrence of phosphorylations within a specific cellular context or cellular response. To identify kinases responsible for the posttranslational regulation of SALL2, we performed bioinformatic analyses using KinasePhos [49], NetPhos 3.1 [50], and ScanSite [51]. This approach identified S763, T778, and S806 phosphorylation residues matching the CK2 consensus sequence (S/T-X-X-D/E) [35]. Phosphorylation of S806 was reported in several studies in co-occurrence with phosphorylation of S802 [52–54]. Although S802 is located within a non-consensus CK2 site (S-X-E), CK2 substrates matching this sequence have been reported [55]. Because CK2-mediated phosphorylation may occur in PEST degradation motifs altering protein stability [44, 45, 56], we evaluated the presence of putative PEST sequences on SALL2 using epestfind [36, 57]. This analysis indicated that the putative CK2 sites are located within or next to a highly conserved putative C-terminal PEST degradation motif (Fig. 1A), close to the DNA-binding domain of SALL2 (ref. [15]). Prediction of structural disorder with IUPred server [58] indicated that the C-terminal PEST motif in SALL2 corresponds to an unstructured solvent-exposed region (Supplementary Fig. 1A), as expected for PEST sequences [59, 60].

To investigate the feasibility of CK2-dependent regulation of SALL2, we analyzed SALL2-CK2α interaction by immunoprecipitation. Endogenous SALL2 mainly interacted with the alpha catalytic subunit of CK2 in HEK 293T cells (Fig. 1B). To further demonstrate this interaction, SALL2-Flag was co-expressed with either HA-tagged CK2α wild-type or HA-tagged CK2α K68M, a previously described dominant-negative mutant unable to bind ATP [60, 61]. Given the transient dynamics of the enzymatic reaction, interaction between overexpressed SALL2 and CK2α was detected mostly when overexpressing mutant CK2α (Fig. 1C). The interaction between SALL2 and CK2α is direct, demonstrated by GST-pulldown using recombinant proteins (Supplementary Fig. 1B).

Next, we conducted in vitro kinase assays. We used monomeric and holo forms of recombinant CK2 [61]. SALL2-Flag was immunoprecipitated from Flip-In^TM^ T-REx^TM^ U2OS cells, followed by a dephosphorylation step with λ-phosphatase. SALL2 was phosphorylated by all forms of CK2, but to a greater extent by CK2α (Fig. 1D, E). Importantly, SALL2 was not phosphorylated by CK2 in vitro unless phosphates were removed with λ-phosphatase prior to the in vitro reaction (Fig. 1D, lanes 5, 9, 13, and 17), suggesting that in vitro phosphorylated residues were already modified under standard cell culture conditions. SALL2 was also phosphorylated without recombinant CK2 (Fig. 1D, lane 1), probably due to the co-immunoprecipitation of endogenous kinases, including CK2. In agreement with this, CK2 inhibition with Silmitasertib, a potent selective and ATP competitive inhibitor of both isoforms of the CK2 catalytic subunits CK2α and CK2α’ [62], decreases the in vitro phosphorylation of immunoprecipitated SALL2 in the absence of recombinant CK2 (Supplementary Fig. 3A). Altogether, our results showed that SALL2 is a CK2 substrate in vitro.

### SALL2 C-terminal PEST motif is phosphorylated in a CK2-dependent manner

**Fig. 2:**
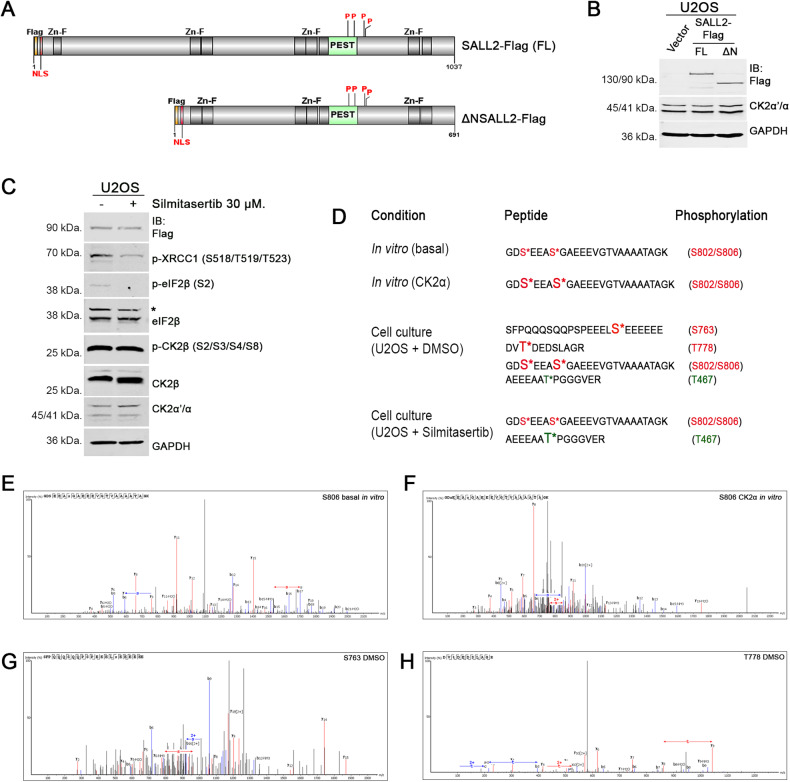
SALL2 C-terminal PEST motif is phosphorylated in a CK2-dependent manner. **A** Scheme of ΔN SALL2-Flag protein lacking most of the N-terminal region, but retaining a putative N-terminal localization signal (NLS). Zinc Finger motifs are highlighted in dark gray and a putative C-terminal PEST motif is shown in green. P, Putative CK2 phosphorylation sites; FL full legth. **B** Ectopic expression of full-length and ΔN SALL2-Flag in Flp-In™ T-REx™ U2OS cells. **C** Assessment of CK2 inhibition after 6 h of treatment with Silmitasertib (CX-4945) at a final concentration of 30 µM in Flp-In™ T-REx™ U2OS cells. Phosphorylation of the CK2 substrates X-Ray Repair Cross Complementing 1 (XRCC1), Eukaryotic Translation Initiation Factor 2 beta (eIF2β), and CK2β in the indicated residues was evaluated [94–98]. Phosphorylation of eIF2β (p- eIF2β) was used as a positive control of CK2 inhibition. *, non-specific band. **D** Phosphorylated peptides identified by Mass Spectrometry after sequential in-gel digestion. In vitro basal, in vitro kinase reaction without addition of recombinant enzyme; in vitro CK2α, in vitro kinase reaction with recombinant CK2α; cell culture (U2OS + DMSO) and cell culture (U2OS+ Silmitarsertib) represent the conditions where SALL2 was immunoprecipitated from DMSO or Silmitarsertib-treated Flp-In™ T-REx™ U2OS cells, respectively. Representative spectra of fragments carrying phosphorylation of S806 after basal (**E**) and CK2α-added (**F**) in vitro kinase reactions are shown. Representative spectra of S763 (**G**) and T778 (**H**)-containing fragments under DMSO treatment show that both residues are phosphorylated under control conditions in Flp-In™ T-REx™ U2OS cells.

We conducted mass spectrometry analyses to identify SALL2 residues phosphorylated by CK2α in vitro or in a CK2-dependent manner in cells. Preliminary experiments led us to conclude that SALL2 C-terminal tryptic peptides are not easily detected using standard mass spectrometry conditions (not shown). In addition, negative charges of acidic residues may lower the ionization efficiency required for conventional positive ion-mode detection [63]. To overcome these issues, we overexpressed and immunoprecipitated a shorter form of SALL2 lacking most of the N-terminal region but retaining an N-terminal putative nuclear localization sequence (NLS) -referred to as ΔN SALL2-Flag (Fig. 2A, B). Next, we performed sequential in-gel digestion with trypsin and Asp-N proteases, as suggested by the MS-Digest tool from protein Prospector to obtain peptides of the appropriate size from the region of interest [64]. The workflow for mass spectrometry is shown in Supplementary Fig. 2A, B.

Like the full-length protein, ΔN SALL2-Flag exhibited nuclear localization and was phosphorylated in vitro by all the different forms of recombinant CK2 (Supplementary Fig. 3). ΔN SALL2-Flag was also phosphorylated without recombinant kinase (Supplementary Fig. 3A, lane 2). Pre-treatment with Silmitasertib impaired this basal phosphorylation (Supplementary Fig. 3A, lane 3), suggesting that ΔN SALL2-Flag is phosphorylated in the absence of recombinant enzyme by co-immunoprecipitated endogenous CK2. In agreement, mass spectrometry results indicated that residues S802 and S806 are phosphorylated without recombinant enzyme. Adding CK2α to the in vitro reaction increased the detection of phosphopeptides carrying these phosphorylations (Supplementary Table 1). These results suggest that S802 and S806 are phosphorylated in vitro by endogenous CK2 and further phosphorylated by the recombinant enzyme.

To determine which sites are phosphorylated in a CK2-dependent manner in cells, transfected Flip-In T-REx U2OS cells were treated with DMSO (vehicle) or Silmitasertib 30 μM for 6 h. CK2 inhibition was confirmed by western blot of known phosphorylated CK2 substrates at specific previously described phosphoresidues (Fig. 2C). ΔN SALL2-Flag was immunoprecipitated, in-gel digested and analyzed (Supplementary Fig. 2B). Our results indicated that all the previously predicted CK2 sites -S763, T778, S802, and S806- are phosphorylated under control (DMSO) treatment, whereas no modified fragments carrying S763 or T778 phosphorylation were detected under Silmitasertib treatment (Fig. 2D, E–H; Supplementary Fig. 2C, D; Supplementary Table 1). Moreover, we identified a novel phosphorylation site corresponding to T467 (Fig. 2D; Supplementary Fig. 2E, F). Unlike phosphorylation of the CK2 putative sites, phosphorylation of T467 was more detectable upon CK2 inhibition. According to the PhosphoMotif Finder tool of HPRD [65], CK1, GSK-3, and/or ERK1/2 might be responsible for this modification. Our results showed that CK2α phosphorylates residues S802 and S806 of SALL2 in vitro, whereas S763 and T778 -located within the putative PEST sequence- are phosphorylated in a CK2-dependent manner under the conditions tested in cells.

### CK2 activity decreases SALL2 stability in cells

**Fig. 3:**
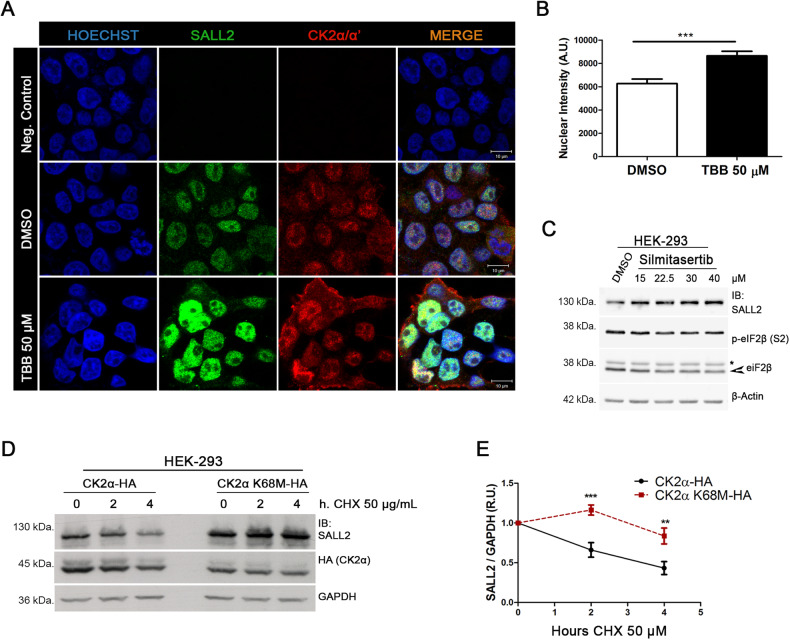
CK2 activity downregulates SALL2 stability. **A** HEK 293 cells were treated with 50 µM of the CK2 inhibitor, TBB. Cells were fixed after 7 h and subjected to immunofluorescence. Endogenous SALL2 is shown in green, whilst endogenous CK2 is stained in red. Both proteins share a nuclear localization. Negative control corresponds to no incubation with primary antibody. **B** Representative graph for the quantification of (**A**), indicating that pharmacological inhibition of CK2 resulted in an augmented immunodetection of SALL2. A.U. arbitrary units; *n* > 100 cells per replicate; error bars equal SEM; ***p < 0.001 (*T*-test). **C** Representative western blot of HEK 293 cells treated for 3 h with various concentrations of the specific CK2 inhibitor, Silmitasertib. **D** SALL2-Flag protein was co-expressed with HA-tagged wild-type CK2α or the K68M dominant-negative mutant in HEK 293 cells. Stability assays were performed using 50 µg/ml of the protein synthesis inhibitor, cycloheximide (CHX), for the indicated time-points. **E** Quantification of SALL2 stability when co-expressed with wild-type CK2α versus the dominant-negative kinase (K68M), normalized against GAPDH. R.U., relative units; error bars represent SEM; **p < 0.01, ***p < 0.001, *n* = 3 (two-way ANOVA plus Bonferroni correction).

**Fig. 4:**
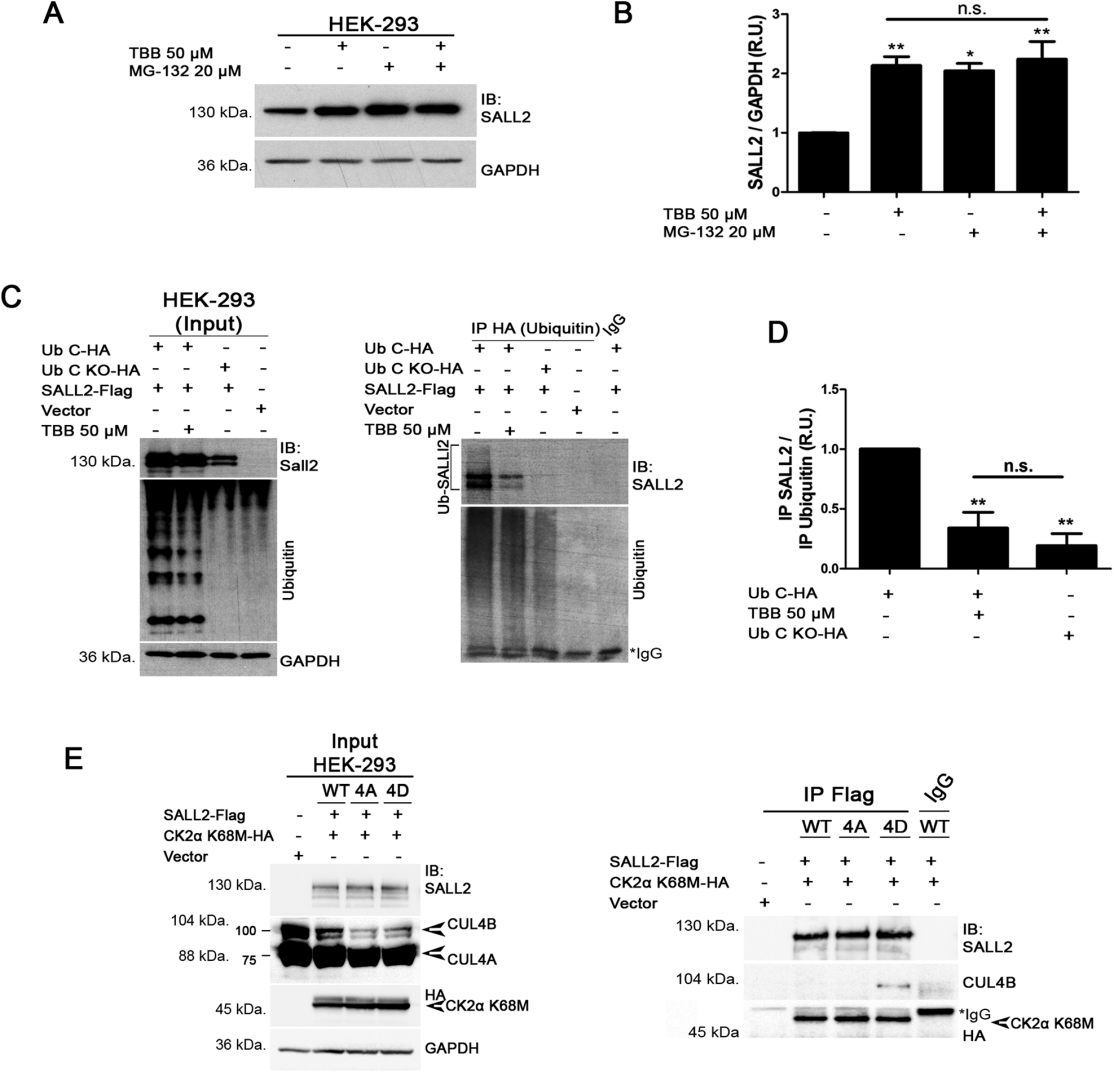
CK2 activity promotes ubiquitylation and proteasome-mediated degradation of SALL2. **A** HEK 293 cells were pretreated with either DMSO (vehicle) or TBB (50 µM) for 3 h, followed by incubation with DMSO (vehicle) or the proteasome inhibitor, MG-132 (20 µM), with or without TBB, for 6 additional hours. Endogenous SALL2 protein levels were quantified and normalized against GAPDH as shown in (**B**). R.U., relative units; error bars equal SEM; *p < 0.05, **p < 0.01, n.s. non-significant *p*-value (one-way ANOVA plus Tukey’s multiple comparison test). **C**, **D** Pharmacological inhibition of CK2 impairs ubiquitylation of SALL2 under proteasome inhibition. SALL2-Flag and Ubiquitin C-HA were co-expressed, and HEK 293 cells were treated as described in (**A**). HA-tagged ubiquitin was immunoprecipitated to enrich the pool of ubiquitylated proteins. SALL2 protein was immunodetected and quantified against Ubiquitin signal. UbC KO-HA, Ubiquitin C with mutated lysine residues; R.U. relative units; error bars represent SEM; **p < 0.01, n.s. non-significant *p*-value (one-way ANOVA plus Tukey’s multiple comparison test). **E** CK2α K68M was co-expressed with either wild-type (WT), non-phosphorylatable (4A), or phosphomimetic (4D) forms of Flag-tagged SALL2 in HEK 293 cells. SALL2 was immunoprecipitated, and interaction with endogenous CUL4B was evaluated. Endogenous CUL4B interacts with phosphomimetic SALL2 protein (4D) but not with the non-phosphorylatable (4A) or wild-type (WT) forms of SALL2.

CK2-mediated phosphorylation can change subcellular localization, stability, and/or activity of its substrates [43–47]. To study whether CK2 impacts SALL2 subcellular localization, we used immunofluorescence to label endogenous and overexpressed proteins. Individual non-phosphorylatable mutant forms of SALL2-Flag exhibited the same subcellular distribution as the wild-type counterpart (Supplementary Fig. 4). Moreover, pharmacological inhibition of CK2 with TBB, a highly selective ATP/GTP-competitive inhibitor of CK2 [66], did not promote changes in the nuclear localization of endogenous SALL2 (Fig. 3A), suggesting that SALL2 subcellular distribution is not affected by CK2-dependent phosphorylation. However, pharmacological inhibition of CK2 with TBB or Silmitasertib increased SALL2 protein levels (Fig. 3B, C). Considering the presence of a putative PEST motif in SALL2, we evaluated whether CK2 activity decreases SALL2 stability. Exogenous proteins were overexpressed to avoid possible transcriptional effects. Stability assays showed that SALL2-Flag is significantly less stable when co-expressed with wild-type CK2α-HA than with the K68M CK2α*-*HA mutant, suggesting that CK2 activity negatively impacts SALL2 protein stability (Fig. 3D, E). We then used HEK293 cells to determine whether CK2 promotes proteasomal degradation of SALL2. Cells were treated with DMSO (vehicle), TBB, the proteasome inhibitor MG-132, or a combination of TBB plus MG-132 (Fig. 4A, B). As previously (Fig. 3A), TBB treatment increased SALL2 protein levels. MG-132 promoted a similar level of SALL2 accumulation. However, combined treatment with the CK2 and the proteasome inhibitors did not result in further accumulation of SALL2 (Fig. 4A, B), suggesting that both CK2 and the proteasome participate within the same pathway. Similar results were observed when overexpressing SALL2 and CK2α, where overexpression of the K68M CK2α*-*HA mutant accounts for CK2 loss of function (Supplementary Fig. 5). To explore further the role of CK2 in the proteasome-dependent degradation of SALL2, we analyzed the ubiquitylation of SALL2 by overexpressing SALL2-Flag and Ubiquitin C-HA in HEK 293 cells. A dominant-negative form of Ubiquitin C (UbC KO-HA) was used as a negative control [67]. Cells were treated with MG-132, and HA-tagged UbC was immunoprecipitated from cell lysates. Ubiquitylated SALL2 was immunodetected in the pool of HA-ubiquitin-labeled proteins (Fig. 4C, D). Our results showed that pharmacological inhibition of CK2 decreases the ubiquitylated SALL2.

Previous reports demonstrated that Cullin 4B ubiquitin ligase (CUL4B) interacts with SALL2, modulating its ubiquitylation in vitro and its degradation under proliferative conditions [68]. Hence, we aimed to explore the effect of CK2-dependent phosphorylation on the interaction of SALL2 with CUL4B. To this end, we mutated all the CK2-dependent phosphorylated residues in SALL2-Flag to Alanine (SALL2-4A). In addition, we generated a phosphomimetic SALL2 mutant in which all the CK2-phosphorylatable residues were substituted by Aspartic Acid (SALL2-4D). Immunoprecipitation assays showed that all the different forms of SALL2 interact with the dominant-negative mutant form of CK2α (CK2α K68M-HA). However, endogenous CUL4B ubiquitin ligase interacted with SALL2-4D but not with the wild-type or 4A form of SALL2-Flag (Fig. 4E) under CK2α K68M-HA overexpression. This result suggests that S763, T778, S802, and S806 phosphorylations are necessary for CUL4B binding. Consistently, SALL2-4A exhibited a trend towards increased stability compared to SALL2 4D or single Alanine mutants (Supplementary Fig. 6C, D) in the H1299 lung cancer cell line. Our data showed that CK2-dependent phosphorylation of SALL2 exerts a negative effect on SALL2 stability by promoting its CUL4B-mediated ubiquitylation and subsequent proteasome-dependent degradation.

### Pharmacological inhibition of CK2 restores SALL2 protein levels in cancer cell lines

**Fig. 5:**
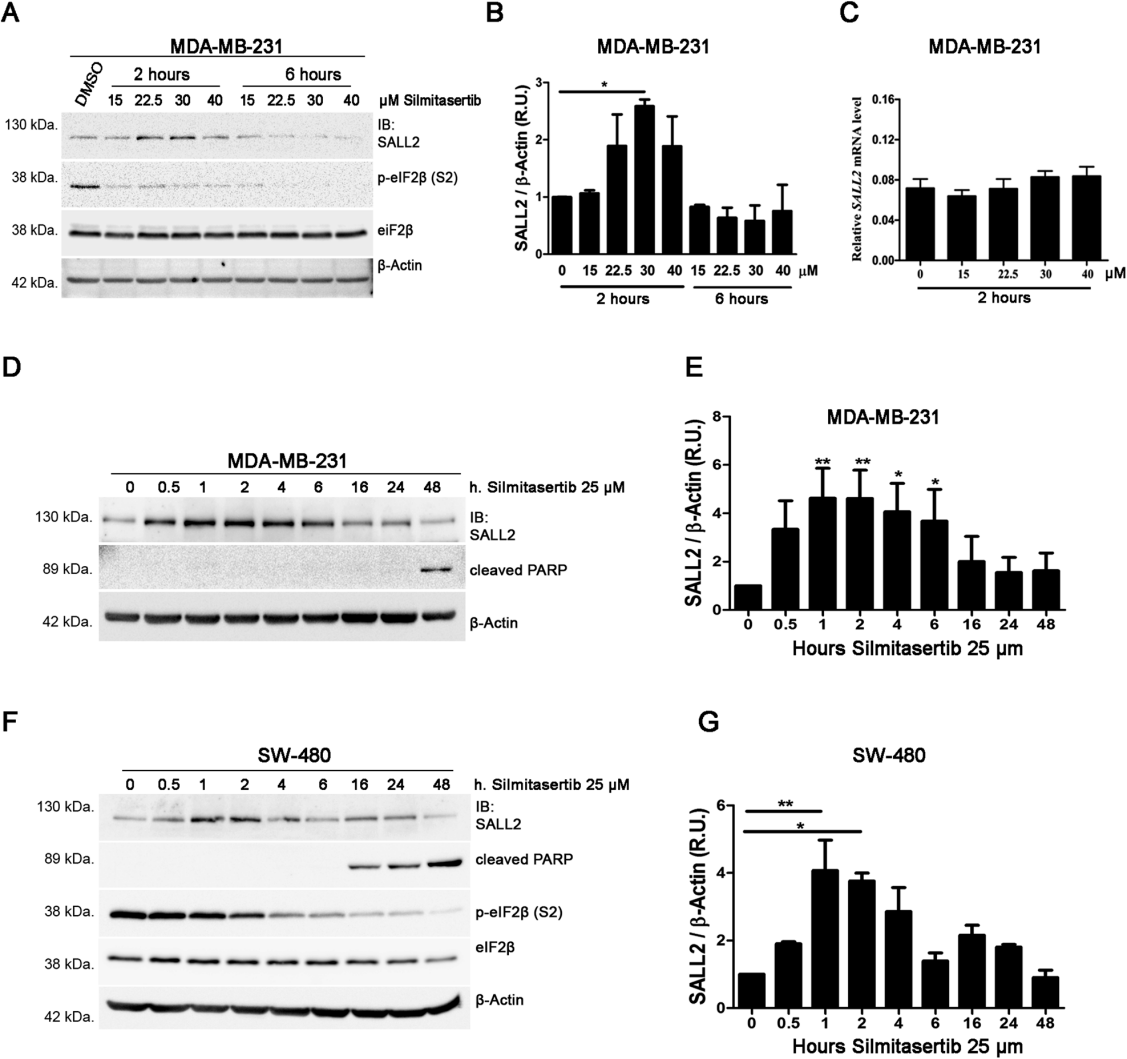
Pharmacological inhibition of CK2 restores SALL2 protein levels in cancer cell lines. **A**, **B** Silmitarsertib increases SALL2 protein levels in MDA-MB-231 triple-negative breast cancer cells. MDA-MB-231 cells were treated with increasing concentrations of Silmitarsertib for two or 6 h. SALL2 protein levels were assessed by western blot (**A**), quantified, and normalized against β-actin (**B**). R.U., relative units; error bars equal SEM; *p < 0.05, (one-way ANOVA plus Tukey’s multiple comparison test). **C** qRT-PCR of *SALL2* normalized to 18S rRNA indicated that augmented transcription is not responsible for the upregulation of SALL2 protein after 2 h of treatment with Silmitasertib at the indicated concentrations. MDA-MB-231 cells (**D**) and SW480 colon cancer cells (**F**) were subjected to CK2 inhibition with Silmitarsertib (25 μM) and collected at the indicated time points. In both cases, SALL2 exhibited a transient upregulation when normalized to β-Actin (**E**, **G**). In addition, cleavage of PARP occurred after long-term treatment with Silmitasertib. R.U., relative units; error bars equal SEM; *p < 0.05, **p < 0.01 (repeated measures ANOVA plus Tukey’s multiple comparison test).

Given that CK2 is overexpressed in cancer [37] and exerts protumoral effects by downregulating tumor suppressors [44, 45], we assessed whether pharmacological inhibition of CK2 via Silmitasertib increases endogenous SALL2 protein levels in different cancer cell lines. Breast MDA-MB-231 and colon SW480 cancer cells were selected based on the fact that there is no reported deletion or transcriptional silencing of the *SALL2* gene -as opposed to SK-OV-3 and HL-60 cell lines [24, 25], suggesting that the selected cells have the capacity to generate the protein product. Before choosing the cells, we confirmed the presence of SALL2 and CK2 proteins in each cell line used (data not shown). The increase in SALL2 protein levels depended on time and treatment concentration between cell lines. MDA-MB-231 human breast cancer cells exhibited a transient increase in SALL2 protein levels under Silmitasertib treatment (Fig. 5A, B, D, E). In contrast, we did not detect changes in mRNA levels (Fig. 5C). Similarly, Silmitasertib transiently increased SALL2 protein levels in SW480 colon cancer cells (Fig. 5F, G, Supplementary Fig. 6A) and H1299 human lung cancer cells (Supplementary Fig. 6B). Long-term treatment resulted in cell death, as shown by immunodetection of cleaved-PARP (Fig. 5D, F). Together, our results suggest that CK2-dependent downregulation of SALL2 occurs in cancer cells.

### Pharmacological inhibition of CK2 induces SALL2-dependent cell death in SW480 colorectal cancer cells and tumor-derived organoids

**Fig. 6:**
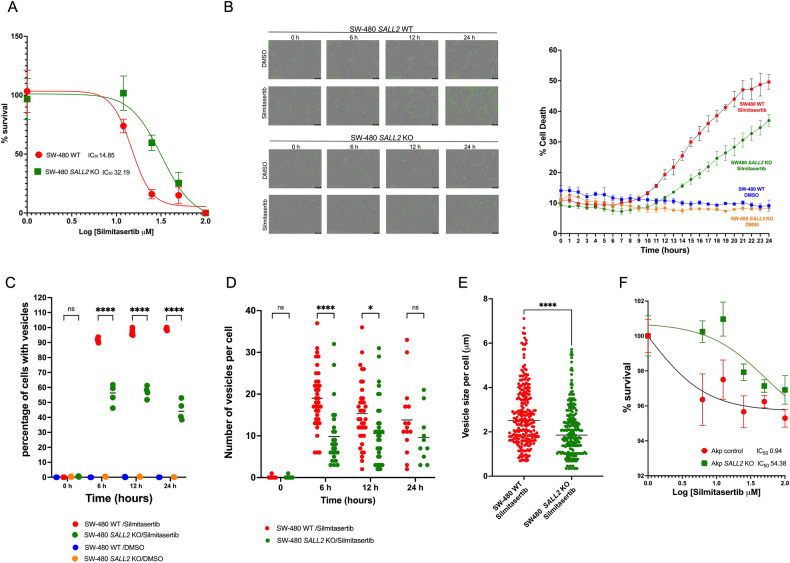
Cytotoxic effects of Silmitarsertib are decreased in SALL2-deficient SW480 cells and Sall2-deficient tumor-derived organoids. **A** IC50 curve for SW480 SALL2 wild-type (clone G10), and SALL2 *knockout* (clone G8) cells treated with Silmitarsertib (0–40 μM) for 24 h. The percentage of cell survival is plotted against the logarithm of treatment concentrations. Data points are the means ± SD of duplicate determinations of triplicate measurements. **B** Real-time cytotoxic assay. The SW480 clones were treated with 25 μM Silmitarsertib and photographed every 1 h for 24 h, using INCUCYTE S3 Live-Cell Analysis System with 20× objective (A representative figure is shown). The dead cells were counted automatically every 1 h for 24 h, and the Cell death percentage was quantified with a cell-by-cell module and normalized by confluence using IncuCyte v2019B software. **C** Percentage of SW480 cells with formation of vacuoles after Silmitarsertib treatment. **D** Analysis of the number of vacuoles per cell. Each point in the graph represents an independent cell. **E** Size of the vacuoles observed after incubation with Silmitarsertib. Each point in the graph represents independent vacuoles observed in the indicated conditions. Analyses of Silmitarsertib-induced vacuoles in **C**, **D**, **E** were performed using ImageJ and Prism GraphPad software, and no less than 100 cells were counted and measured per condition. **F** IC50 curve for AKP control and AKP SALL2 *knockout* tumor-derived organoids treated with Silmitarsertib (0-100 μM) for 24 h. The percentage of cell survival is plotted against the logarithm of treatment concentrations. Data points are the means ± SD of duplicate determinations of triplicate measurements.

Given that SALL2 promotes cell death [6], we reasoned that the downregulation of SALL2 by CK2 may impact cell survival in cancer cells. To address whether the tumor-suppressive role of SALL2 mediates cell death under treatment with Silmitasertib, we used the SW480 cells, which express SALL2 and overexpress CK2α [69, 70]. We generated a *SALL2* knockout (*SALL2* KO) SW480 cell model by CRISPR/Cas9 genome editing as previously [71] (Supplementary Fig. 7). Initially, we evaluated IC50 in isogenic SW480 *SALL2* wild-type (WT) and *SALL2* KO cells subjected to Silmitasertib (0-40 μM) treatment for 24 h. The surviving fraction and IC50 in response to Silmitasertib were significantly higher in *SALL2* KO cells (2.16-fold) than in SW480 wild-type cells (Fig. 6A). Next, we treated cells with Silmitasertib (25 μM) in the presence of 30 nM Sytox Green to evaluate real-time live-cell cell death assay over a 24-h exposure period. SW480 *SALL2* KO cells showed a significant delay and overall decrease in the percentage of cell death induced by Silmitasertib than SW480 *SALL2* WT cells, indicating that SALL2 sensitizes SW480 cells to Silmitasertib cytotoxicity (Fig. 6B, Supplementary video 1 and 2). Interestingly, we also noticed a massive formation of vesicles at early time points (4-6 h.) that preceded Sytox staining, particularly in *SALL2* WT cells compared to *SALL2* KO cells. Previous reports have shown that Silmitasertib results in a non-apoptotic cell death program known as methuosis [70, 72], characterized by increased cytoplasmic vacuolization or macropinosomes [72–74]. We evaluated the percentage of cells with vesicles, the number of vesicles per cell, and the size between *SALL2* WT and *SALL2* KO cells in response to the Silmitasertib. Silmitasertib significantly increased the percentage of cells with vesicles and the number of vesicles per cell in both cell lines (Fig. 6C). However, SW480 *SALL2* KO cells showed a lower percentage of cells with vesicles and fewer vesicles per cell at 6- and 12-h treatment than the *SALL2* WT cells (Fig. 6D). Moreover, the vesicle size per cell induced by Silmitasertib in SW480 *SALL2* KO was smaller than in the wild-type cells (Fig. 6E). These results indicated that the methuosis-like phenotype induced by Silmitasertib depends on SALL2 expression. Additional morphologic features associated with a methuotic phenotype include cell swelling, lamellipodia or ruffle membrane projections, and membrane rupture [48]. Previous studies have shown that even though caspase and PARP activation may concomitantly occur with methuotic cell death, this is not an obligatory step, and no pyknotic nuclei are observed in cells with severe vacuolization [73, 74]. Accordingly, our results showed that Silmitasertib treatment results in PARP cleavage in SW480 cells after 16 h of treatment and in MDA-MB-231 breast cancer cells after 48 h of treatment (Fig. 5F, D), but the massive vacuolization is induced as early as 4 h of exposure to Silmitasertib in SW480 wild-type cells (Supplementary Video 1). To assess additional morphological features, we evaluated actin rearrangement and nuclei integrity in cells treated with Silmitasertib. Nuclei and actin filaments were stained with DAPI and Phalloidin-iFluor™ 488, respectively. Consistent with previous reports [74, 75], both cell lines showed relatively intact nuclei, but SW480 *SALL2* WT exhibited more plasmatic vacuolization with peri-vesicular actin compared to a significantly lower number of vesicles, smaller vesicles per cell, and fewer actin clusters of *SALL2* KO cells after 24 h of treatment with Silmitasertib treatment (Supplementary Fig. 8).

We next conducted experiments involving mouse tumor organoids to investigate the relevance of SALL2 expression on the Silmitasertib cytotoxicity in a more physiologically relevant context. Following the above studies in the 2D culture from SW480 colorectal cancer cells, we generated tumor organoids from a genetically engineered mouse model carrying *villin*Cre^ER^, *Apc*^fl/fl^, *Kras*^G12D/+^, *p53*^fl/fl^ (AKP), which recapitulates key genetic changes observed in human colorectal cancer [76, 77]. AKP-Sall2KO organoids were generated by CRISPR/Cas9 genome editing and validated via sequencing and western blot (Supplementary Fig. 9). We treated the AKP (non-targeting control) and the AKP-Sall2KO organoids with different concentrations of Silmitasertib (0–100 μM) and cell viability was measured 24 h post treatment. The cutoff value of the half-maximal inhibition concentration (IC50) for the Silmitasertib sensitivity was significantly lower in the AKP organoids than the AKP-S2KO (IC_50_AKP = 1.798 vs. IC_50_AKP-S2KO = 54.38) (Fig. 6F), confirming that *Sall2*-deficient cancer cells are more resistant to Silmitasertib-dependent cell death. Overall, our results show that *SALL2/Sall2* expression sensitizes cancer cells to Silmitasertib cytotoxicity, indicating that restoration of SALL2/Sall2 function favors cell death in response to CK2 inhibition.

## Discussion

**Fig. 7:**
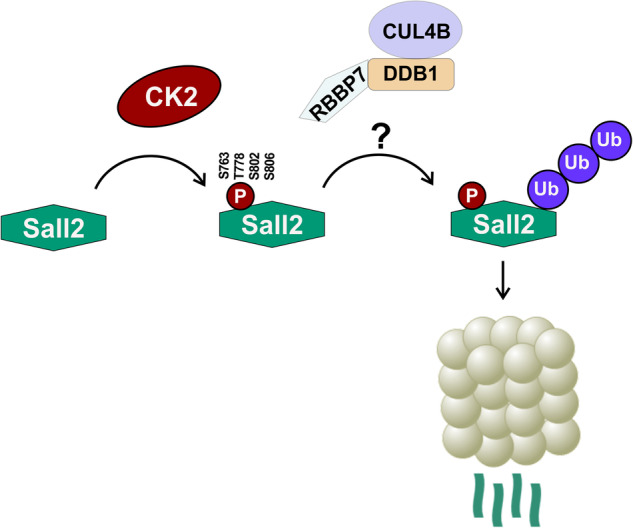
Proposed model for CK2-dependent regulation of SALL2. CK2 activity promotes the phosphorylation of SALL2 in S763, T802, S802 and S806. Phosphorylated SALL2 interacts with CUL4B, a component of the Cullin-Ring Ubiquitin-Ligase Complexes (CRLs), being subjected to ubiquitylation followed by proteasomal degradation. Pharmacological inhibition of CK2 impairs this process, triggering the accumulation of SALL2 in cancer cells.

Understanding mechanisms involved in regulating transcription factors’ activity is essential because of their role in gene expression and, ultimately, in cell behavior. SALL2 is a transcription factor involved in cell development, cell cycle arrest, apoptosis, cell migration, and disease [14, 16, 19, 21, 78]. SALL2 is downregulated or upregulated in cancer depending on the cancer type and stage. Nonetheless, mechanisms regulating SALL2 expression remain poorly understood. Our work identified SALL2 as a novel CK2 substrate. CK2 directly phosphorylates SALL2 residues S763, T778, S802, and S806, inducing its proteasomal degradation (Fig. 7, model). To our knowledge, this is the first report identifying a kinase that post-translationally modifies SALL2.

Bioinformatic analyses revealed residues S763, T778, and S806 as putative phosphorylation sites for CK2 at the C-terminal region of SALL2. Mass spectrometry analyses identified that CK2α phosphorylates these three residues and S802. However, we found discrepancies between the CK2α -dependent SALL2 phosphorylated residues identified in vitro and those in cells. We determined that the S763 and T778 phosphorylation depend on CK2 activity but only occurs in cells, suggesting that their regulation by CK2 is either mediated by a different CK2 isoform (the holoenzyme or CK2α’ as opposed to CK2α) or requires other proteins and/or cell conditions. On the other hand, we found that SALL2 exhibited basal phosphorylation in the absence of recombinant CK2 kinase, indicating that in vitro phosphorylated sites in SALL2 are already modified in cultured cells. The basal phosphorylated sites correspond to S802 and S806, suggesting that endogenous CK2α mediates their constitutive phosphorylation in cells. The detection of S802 and S806 phosphopeptides when the protein is immunoprecipitated from cells treated with Silmitasertib may be explained by (1) insufficient inhibition of CK2 under the conditions tested in this study and/or higher affinity of CK2 for these sites or (2) that these sites might also be a substrate for other acidic kinases such as GSK-3, e.g., as part of a hierarchical phosphorylation mechanism where CK2 primes for further GSK-3-mediated phosphorylation, as it has been reported for Glycogen Synthase [79]. Previously, phosphoproteomic studies found SALL2 phosphorylated at S802 and S806 under various conditions in different cell models and proposed CK2 as a potential kinase [52–54, 80, 81]. GSK-3 was alternatively proposed for S802 phosphorylation [53, 79]. Even though there is currently no evidence of SALL2 regulation by GSK-3, this possibility can not be excluded and requires additional studies.

Constitutive CK2-dependent phosphorylation of proteins is not uncommon. IκBα and STAT3 are constitutively phosphorylated by CK2 (ref. [47, 82]). Interestingly, the constitutive STAT3 phosphorylation specifically occurs in chronic lymphocytic leukemia (CLL) cells, contributing to its transcriptional function and cell survival and proliferation [82]. The phosphorylation of IκBα occurs in a C-terminal PEST domain and affects the unstimulated turnover of the protein [47]. A potential carboxyl terminal PEST sequence in SALL2 locates in the same region where we found the CK2 phosphorylation sites. In this context, analysis of SALL2 protein levels in the presence of TBB or catalytically inactive CK2α K68M showed that CK2 decreases the stability of SALL2 in a ubiquitin-proteosome-dependent manner.

Because the S802 and S806 residues are located next to the C-terminal PEST motif of SALL2, we could expect that their phosphorylation affects SALL2 stability and function. However, mutation of S802 or S806 to non-phosphorylatable alanine did not significantly affect SALL2 stability. We cannot exclude that phosphorylation of both residues is required to affect SALL2 stability. Instead, they may serve to prime SALL2 for subsequent stimulated degradation by phosphorylation of the other residues within the PEST motif. In agreement, mutations of all the CK2-dependent residues to alanine, including S763 and T778 within the PEST motif, stabilized SALL2. In contrast, mutations of all of them to aspartic acid resulted in SALL2 interaction with the CUL4B ubiquitin ligase. Because SALL2 has tumor suppressor activity in some cancer types, CK2 may contribute to tumorigenesis by inducing SALL2 degradation.

CK2 activity, expression, or both are elevated in normal tissues with a high mitotic index and various cancers, including leukemia, head and neck, prostate, kidney, mammary gland, and lung [83, 84]. CK2 promotes tumorigenesis by activating oncogenes and negatively regulating tumor suppressors [83]. CK2 can affect its targets’ subcellular localization, stability, and activity [43–47]. Our study suggests that CK2 targets SALL2 for degradation. The identified CK2-SALL2 axis may be part of a broader mechanism where CK2 downregulates the stability of multiple tumor suppressors in the cell, including iκB, VHL, PML, and SALL2, by phosphorylating residues near or within PEST motifs [44, 45, 47].

What cellular context favors SALL2 degradation by CK2 is unknown. Associated with its role in tumorigenesis, CK2α is phosphorylated in several residues at the beginning of mitosis by CDK1 kinase. The phosphorylation of CK2 regulates its localization in the mitotic spindle and is critical for the cycle progression [85]. Our previous studies showed that SALL2 levels fluctuate during the cell cycle, being high in mitosis and decreasing as the cycle progressed toward G1. Because endogenous and overexpressed SALL2 suffered similar fluctuation in their expression during the cell cycle, it suggested that SALL2 is post-translationally regulated [14, 68]. Since CK2 targets several substrates during the cell cycle, it is possible that CK2 targets SALL2 for proteasome degradation during G1 entry. Ubiquitin-ligase complexes of the culin family play an important role in the degradation of phospho-proteins during the cell cycle [86]. In agreement with its posttranslational regulation and degradation during the cell cycle, SALL2 is ubiquitinated by the CUL4B-DDB1-RBBP7 complex. Subsequently, the proteasome degrades it, which induces the proliferation of human fibroblasts in a quiescent state [68]. However, the potential regulation of SALL2 by CK2 during the cell cycle requires further studies, which is out of the scope of this report.

Previous studies have shown that the SALL2 ortholog in *Drosophila melanogaster*, SALM, is a CK2 interactor [87], suggesting that the described mechanism is conserved across species. However, the highly conserved identified sequence containing putative CK2 phosphorylation sites and C-terminal PEST motif is only present in SALL2 and not conserved in any paralog in mammals (not shown), adding the difference between SALL2 and SALL1, 3, and 4 members’ regulation.

Treatment with Silmitasertib increased the levels of SALL2 in H1299, MDA-MB-231, and SW480 cancer cell lines. However, this effect was not observed in SK-OV-3 ovarian cancer cells, where SALL2 silencing occurs at the transcriptional level by hypermethylation of the P2 promoter [24]. Neither in the human acute promyelocytic leukemia cell line, HL -60, consistent with the reported absence of *SALL2* transcript in human acute myeloid leukemias [25], Supplementary Fig. 10). These results indicate that CK2- dependent SALL2 regulation depends on the cancer cell context, genetic and/or epigenetic alterations, which may affect Silmitasertib treatment efficacy. Additionally, it is worth noting that Silmitasertib and TBB may also modulate other unidentified proteins with distinct functionalities compared to SALL2.

We observed that Silmitasertib induced a methuosis-like phenotype, with the appearance of cytoplasmic vacuoles of different sizes in SW480 cells, whereas loss of *SALL2* delayed and ameliorated this phenotype. Events related to non-apoptotic cell death by vacuolization, such as methuosis, are diverse and not fully understood. Still, the methuosis phenotype includes the loss of cell viability preceded by extreme cytoplasmic vacuolization and occurs without the classic morphological features of apoptosis. Even though Caspase and PARP cleavage concomitantly occur, they are not required for the loss of viability caused by massive vacuolization [74, 75]. Consistent with this statement, the massive vacuolization was evident as early as 4 h after treatment with Silmitasertib in SW480 wild-type cells. In comparison, PARP cleavage was not observed until 16 h after treatment. *SALL2* knockout by CRISPR-Cas9 results in lower sensitivity to Silmitasertib than control cells, accompanied by fewer and smaller cytoplasmic vesicles. Our results indicated that pharmacological inhibition of CK2 could induce multiple types of cell death. Those mechanisms may be related to the CK2-dependent regulation of SALL2 and require further investigation.

Here, we have demonstrated that SALL2 increases the Silmitasertib cytotoxicity in SW480 colorectal cancer cells and in AKP-derived organoids, a valid alternative to animal treatment that supports the relevance of the association between CK2 and SALL2. SALL2 is also critical for the cytotoxic effects of genotoxic chemotherapeutic drugs such as doxorubicin and etoposide [15, 16, 22] and necessary for the cellular-apoptotic response to chemotherapeutics independently of p53 [6]. Thus, therapies combining agents that induce DNA damage with Silmitasertib may promote synergistically SALL2-dependent cancer cell death. However, it is important to consider that the *SALL2* locus has been reported to undergo LOH in certain types of cancer [28–30], likely compromising the efficacy of these combined treatments. Further research is needed to identify other genes involved in promoting methuosis-like phenotype in response to Silmitasertib or conditions sensitizing cells to undergo methuosis, such as RAS hyperactivation [75, 88, 89]. Thus, those studies may predict the best therapeutic strategies based on specific tumoral molecular features.

We have shown that SALL2 upregulation is a specific response to CK2 inhibition because 1) SALL2 protein levels increase not only in response to Silmitasertib but also by overexpressing a dominant-negative form of CK2 and in response to TBB [66]. 2) SALL2 and CK2 interact directly. 3) SALL2 is phosphorylated by CK2 in vitro, and 4) phosphomimetic SALL2 mutant shows increased interaction with a E3 ubiquitin ligase complex that contains CUL4B. In conclusion, our work identified SALL2 as a novel CK2 substrate and demonstrated that CK2 activity negatively regulates SALL2 stability. This report constitutes the first study to describe a phosphorylation-mediated regulatory mechanism of SALL2. We propose a new mechanism for reducing SALL2 levels in cancer cells, which may have implications for cancer therapy with CK2 inhibitors.

## Methods

### Reagents

Dulbecco’s modified Eagle’s medium (DMEM), Roswell Park Memorial Institute (RPMI-1640), and Fetal Bovine Serum (FBS) were obtained from Hyclone (Cytiva, Marlborough, MA, USA). DMEM without phenol red (Cat. # 01-053-1A) was from Biological Industries (Sartorius Group, Kibbutz Beit-Haemek, Israel). l-Glutamine, Penicillin/Streptomycin and lipofectamine 2000 were from Thermo Fisher Scientific (Waltham, MA, USA). Isopropyl-β-D-1-thiogalactopyranoside (IPTG), Colloidal Coomassie G-250, Z-Leu-Leu-Leu-al (MG-132) and 4,5,6,7-Tetrabromobenzotriazol (TBB) were purchased from Sigma-Aldrich (San Luis, MO, USA). Silmitasertib (CX-4945) was from MedChemExpress (Monmouth Junction, NJ, USA). Dimethylsulfoxide (DMSO), Cycloheximide (CHX), Protein AG Plus-agarose resin (Cat. # SC-2003) were obtained from Santa Cruz Biotechnology (Dallas, TX, USA). Protein G-Sepharose®4 Fast Flow resin (Cat. # 17-0618-01) and ECL reagent were from General Electric Healthcare (Chicago, IL, USA). PVDF membranes (Immobilon; Millipore) and Horseradish Peroxidase (HRP)-conjugated antibodies were purchased from Merck (Kenilworth, NJ, USA) and BioRad (Hercules, CA, USA), respectively. Secondary antibodies conjugated with Alexa Fluor 488 and Alexa Flour 555 were from Invitrogen (Thermo Fisher Scientific) and Hoechst 33342 stain (Cat. # 639) was from ImmunoChemistry Technologies (Bloomington, MN, USA). Primary antibodies to detect CK2α-only, CK2α’ (GST-fusion), CK2β (KLH), phospho-CK2β (S2/S3/S4/S8), phospho-eIF2β (S2), as well as plasmids pRCCMV/CK2α-HA and pRCCMV/CK2α(K68M)-HA were provided by Dr. David W. Litchfield (61). Anti-HA (Cat. # MMS-101P), anti-6xHis (Cat. # 631212) and anti-eIF2β (Cat. # GTX106484) antibodies were from Covance (Princeton, NJ, USA), Clontech (Mountain View, CA, USA), and Genetex (Irvine, CA, USA), respectively. Antibodies against SALL2 (Cat. # A303-208A) and phospho-XRCC1 (S518/T519/T523) (Cat. # A300-059A) were obtained from Bethyl Laboratories (Montgomery, TX, USA). Anti-FLAG (Cat. # F3165), anti-CK2α/α’ (Cat. # C5367) and anti-SALL2 (Cat. # HPA004162) were from Sigma-Aldrich. Anti-CK2α/α’ (Cat. # 05-1431) and anti-GAPDH (Cat. # MAB374) were purchased from Merck (Darmstadt, Germany). Anti cleaved-PARP (Cat. # SC-56196), CUL4 (H-11, Cat. # SC-377188), β-Actin (Cat. # SC-69879), Ubiquitin (Cat. # SC-8017), CK2β (6D5, Cat. # SC-12739) and β-Catenin (Cat. # SC-1963) were from Santa Cruz Biotechnology. The KAPA HiFi HotStart ReadyMix PCR Kit was from Roche Molecular Systems, Inc. All restriction enzymes were purchased from New England Biolabs (Ipswish, MA, USA).

### Cell culture

HEK293 (ATCC Cat# CRL-1573), HEK293T (ATCC Cat# CRL-3216™), MDA-MB-231 (ATCC Cat# HTB-26), and Flp-In™ T-REx™ U2OS cells (kindly provided by Dr. Karmella Haynes, Arizona State University) were cultured in DMEM Hyclone (Cytiva, Marlborough, Massachusetts, USA) with 10% fetal bovine serum (FBS), L-Glutamine, Penicillin/Streptomycin (Thermo Fisher Scientific). H1299 (ATCC® CRL-5803™), SW480 (ATCC Cat# CCL-228), and HL-60 cells (ATCC® CCL-240™, kindly gifted by Dr Soraya Gutiérrez, Universidad de Concepción) were grown in RPMI-1640 (Hyclone) supplemented with 10% FBS, L-Glutamine, and Penicillin/Streptomycin. SK-OV-3 cells (ATCC® HTB-77™, kindly provided by Dr. Gareth Owen, (Pontificia Universidad Católica de Chile) were cultured in DMEM-F12 media (Gibco, Thermo Fisher Scientific) with 10% FBS, L-Glutamine, and Penicillin/Streptomycin. All cell lines were kept at 37 °C in a humidified incubator with 5% CO_2_. When indicated, cells were treated with CHX, MG-132, TBB, and/or Silmitarsertib (CX-4945), using DMSO as a vehicle.

Cancer cell lines authentication was done by molecular genetic Short Thandem Repeat (STR) loci analysis, performed by AMP-FLP (Amplified Fragment Length Polymorphism) using the PowerPlex® Fusion System (Promega Corporation, Madison, WI, USA) and electrophoretic separation of the alleles in an automatic genetic analyzer model ABI 310, Applied Biosystems (ThermoFisher Scientific).

Cell cultures were routinely tested for mycoplasm using the EZ-PCR MycoplasmaTest kit from Biological Industries.

### Cell transfection

Transient transfection of cell lines was performed using calcium phosphate [90], except for H1299 cells, where lipofectamine 2000 was used following the manufacturer’s directions.

### Generation of CRISPR-Cas9-mediated *SALL2* knockout SW480 cancer cells

SW480 *SALL2* knockout clones (SW480 G8 and SW480 G4) were obtained by CRISPR-Cas9, as described in [71]. Briefly, SW480 cells were electroporated at 1300 V per 20 ms (NEON Transfection System, Thermo Fisher Scientific), with a vector encoding CRISPr/Cas9 coupled to Paprika-RFP (ATUM Bioengineering Solutions, www.atum.bio) and harboring the specific *SALL2* guide RNA [71]. Cells were sorted by RFP channel (BD FACSAria III cell sorter, BD Biosciences; San Jose, CA, USA.) and plated as individual clones. For knockout identification, a western blot against SALL2 and genomic PCR were performed on each clone. A similar procedure was followed to obtain the SW480 *SALL2* WT control clones. Selected positive clones were further confirmed by sequencing analysis at Pontificia Universidad Católica Sequencing Facility, Santiago, Chile.

### Plasmids

SALL2 mutants were obtained by site-directed mutagenesis of pCMV2(NH)/SALL2-Flag using the KAPA HiFi HotStart ReadyMix PCR Kit. SALL2-Flag S763, T778, S802, and S806 residues were sequentially substituted by alanine or aspartic acid. Primers used for mutations are indicated in Supplementary Table 2. To generate the ΔNSALL2-Flag construct, pCMV2(NH)/SALL2-Flag was digested with PpuMI to remove the sequence encoding aminoacids 32-377 while retaining a putative N-terminal nuclear localization signal of SALL2. For recombinant SALL2-His (full length, FL), pCMV2(NH)/SALL2-Flag was digested with BstUI, and the released *SALL2* sequence was subcloned into the pQE81-L vector previously linearized with SmaI. Subcloning and site-directed mutagenesis of *SALL2* were confirmed by sequencing analysis at Pontificia Universidad Católica Sequencing Facility, Santiago, Chile. pRCCMV/CK2α-HA (Addgene plasmid #27086), pRCCMV/CK2α K68M-HA (Addgene plamid. #27089), and pGEX 3X-CK2α-GST were provided by Dr. David W. Litchfield [61]. pRK5/UbC-HA (Addgene plasmid #17608) and pRK5/UbC KO-HA (Addgene plasmid #17603) were kindly provided by Dr. Ted Dawson (Addgene plasmids #17608 and #17603).

### Purification of SALL2-His recombinant protein

*Escherichia coli* BL-21 were transformed with pQE81-L/hSall2 FL-His and treated with 500 μM of IPTG to induce recombinant protein expression. Purification of SALL2 FL-His was conducted by using Nickel-Ni-NTA agarose resin (Qiagen, Cat. No. # 1018244). Ni-NTA resin was deposited on a Poly-Prep® column (BioRad) and the protein of interest was eluted with lysis and wash buffer (NaH_2_PO_4_ 50 mM, NaCl 300 mM, Imidazole 20 mM) supplemented with increasing concentrations of Imidazole: 100–150–200–250 mM. The protein elutions obtained were filtered in an Amicon® Ultra-4 filtration unit of 100 K MWCO (Molecular Weight Cut-Off, Millipore). The band corresponding to hSALL2-His was identified by mass spectrometry at the MALDI Mass Spectrometry Service of the London Regional Proteomics Center at the University of Western Ontario, Canada.

### GST-pulldown

*E. coli* BL-21 CodonPlus bacteria were transformed with pGEX 3X-CK2α-GST (pDB1) or pGEX-4T-3 [61]. Then, IPTG was added to induce the expression of recombinant proteins. For GST-proteins purification, Glutathione-Sepharose 4B resin (Bioworld#201820032) was used. GST- and GST-CK2α- loaded resins were incubated with purified hSALL2-His for 12 h in Igepal buffer (Tris 50 mM, pH 8.0; NaCl 150 mM; MgCl_2_ 5 mM; Igepal 1% v/v; sodium deoxycholate 0.25% w/v; glycerol 10% v/v) after which the resin was washed four times with 1× Igepal buffer. Finally, 2× loading buffer was added, and protein-protein interaction was analyzed by western blot.

### Western blot

Cells were harvested and lysed in lysis buffer (Tris pH 7.5, 50 mM; NaCl 150 Mm, MgCl_2_ 2.5 mM; Triton 1%; Glycerol 10%) supplemented with protease and phosphatase inhibitor cocktails (Sigma-Aldrich). Protein cell lysates were separated by SDS-PAGE and transferred to PVDF membranes. When indicated, primary antibodies against CK2α’/α, CK2, CK2β, CUL4A, CUL4B, β-Actin, cleaved-PARP, eIF2β, GAPDH, p-CK2β (S2/S3/S4/S8), p-XRCC1 (S518/T519/T523), p-eIF2β (S2), SALL2, Ubiquitin, FLAG, HA and 6xHis, were used. After incubation with HRP-conjugated secondary antibodies, membranes were treated with ECL reagent and exposed in Syngene’s Multi-application gel imaging system PXi for protein detection. All quantifications were conducted using ImageJ using non-edited uncropped images [91], normalized against loading controls.

### Immunoprecipitation

HEK293 cells were transfected with one of the indicated plasmids: pCMV2(NH)/SALL2-Flag, pCMV2(NH)/SALL2 S763/T778/S802/S806A-Flag (mutant SALL2 4A-Flag), pCMV2(NH)/SALL2 S763/T778/S802/S806D-Flag (mutant SALL2 4D-Flag), together with one of the following plasmids: pRCCMV/CK2α-HA and/or pRCCMV/CK2α K68M-HA (inactive mutant), pRK5/UbC-HA, or pRK5/UbC KO-HA (inactive mutant). Protein cell lysate (0.5–2 mg) were pre-cleared with resin before adding the corresponding primary antibody (Anti-HA or Anti-Flag) for 1 h. Then, Protein AG Plus-agarose was added to the samples and incubated overnight. Finally, to remove the supernatant, samples were washed, and 30 μL of 2× loading buffer was added to the immunoprecipitated. The interaction between proteins was analyzed by western blot. For immunoprecipitation of endogenous SALL2, anti-SALL2 antibody (Bethyl, Cat. # A303-208A) was used.

### In vitro phosphorylation assays

Flp-In™ T-REx™ U2OS cells were transfected with pCMV(NH)/SALL2-Flag or pCMV(NH)/ΔNSALL2-Flag and lysed with TLB buffer (NaCl 150 mM; Tris-HCl 50 mM, pH 7.5; MgCl_2_ 2.5 mM; Igepal 1%; sodium deoxycholate 0.1%; glycerol 10%; supplemented with 1 mM PMSF; 7 µg/mL pepstatin A; 20 µg/mL leupeptin; 2.9 µg/mL aprotinin) in the absence of phosphatase inhibitors. Then, protein cell lysate was pre-cleared with resin, and 2.5 µg of anti-Flag antibody was added. After incubation overnight, 30 μL of Protein G-Sepharose®4 Fast Flow resin was added and incubated for 2 h to immunoprecipitate SALL2. Recombinant proteins on the beads were washed several times with TLB buffer and reserved for phosphorylation assays.

To perform the in vitro phosphorylation assays, immunoprecipitated SALL2-Flag on the beads was resuspended in metalophosphatase buffer (PMP, New England Biolabs, Cat. No. # B0761S) with 1 mM MnCl_2_. Then, recombinant λ-Phosphatase was added at 30 °C for 40 min before stopping the reaction with TLB buffer (with 1 mM Na_3_VO_4_). For enzymatic kinase reaction, immunoprecipitated SALL2-Flag was mixed with 13 U/mL of recombinant CK2 (CK2α; CK2α′; CK2α2β2 and CK2α′2β2 purified from bacteria) [92] in 5× reaction buffer (250 mM Tris (pH 7.5); 750 mM NaCl; 50 mM MgCl_2_; 0.5 mM ATP; 100–200 μCi/mL [γ^32^]-ATP). The kinase reaction was incubated for 10 min at 30 °C with stirring and was stopped by adding of 3X loading buffer. When indicated, control reactions were pre-incubated with 100 nM Silmitarsertib (without 5× reaction buffer) for 10 min on ice prior to phosphorylation assays. The samples were subjected to polyacrylamide gel electrophoresis and stained with 0.1% v/v of Colloidal Coomassie G-250 solution. Finally, the gel was dehydrated and exposed to an autoradiographic film overnight. The signal bands obtained were quantified with ImageQuant Version 5.2 software (Molecular Dynamics).

### Mass spectrometry

ΔNSALL2-Flag was immunoprecipitated from Flp-In™ T-REx™ U2OS cells transfected with pCMV(NH)/ΔNSALL2-Flag, treated with DMSO or 30 μM Silmitasertib for 6 h. For mass spectrometry analyses of in vitro phosphorylated SALL2, cold ATP was added to the 5X reaction buffer for in vitro phosphorylation assays described above.

Samples were subjected to polyacrylamide gel electrophoresis followed by gel staining and extraction with the MASSPrep Automated Digestor (Waters/Micromass) from the London Regional Proteomics Center at the University of Western Ontario, Canada. After removing the dye, immunoprecipitated ΔNSALL2-Flag was digested with Trypsin in 50 mM NH_4_HCO_3_ solution, followed by digestion with Asp-N (Sigma-Aldrich). Peptides obtained from digested samples were extracted from the gel and sent to London Regional Proteomics Center for Mass Spectrometry analyses. The samples were subjected to tandem mass spectrometry in the Q-tof Ultima Global equipment (Micromass), with electrospray ionization (ESI) and fragmentation by activated/collision-induced dissociation (CAD/CID). The analyses were performed in PEAKS Studio software [93] in the UniProt database for Homo sapiens, with a 1% FDR (false discovery rate).

### Reverse Transcription-quantitative PCR

Total RNAs were extracted from cells with Trizol reagent (Life Technologies Inc.) according to the manufacturer’s instructions and coupled with RNA purification columns (Omega Biotek, USA). Before qPCR, the RNA was treated with Turbo DNase (Ambion) to eliminate any residual DNA from the preparation. One microgram of the total RNA was reverse transcribed using the M-MLV reverse transcriptase (PROMEGA) and 0.25 μg of Anchored Oligo(dT) 20 Primer (Invitrogene). qPCR was performed using KAPA SYBR FAST qPCR Master Mix Kit and the AriaMX Real-Time PCR System (Applied Biosystem, USA) according to the manufacturer’s instructions. Thermal cycling variables used were as follows: 40 cycles at 95 °C for 10 s and 60 °C for 20 s. Amplification of SALL2 was assessed using the following nucleotides generating a 189 bp fragment: forward 5′-TATGTGCTAGAGCCCTTGGGG-3′; reverse 5′-CACTCGGAGACAGATGACA-3′. Amplification of 18S was assesed for each sample as an endogenous control, using the following nucleotides generating a 100 bp fragment: forward 5′-GTAACCCGTTGAACCCCATT-3′; reverse 5′-CCATCCAATCGGTAGTAGCG-3′. The relative expression ratio of each gene was calculated using the standard curve method, using untreated (vehicle) cells as a reference. Expression of SALL2 was relative to 18S.

### Cytotoxic assay and Silmitarsertib IC50 determination in SW480 colorectal cancer cells

IC_50_ of Silmitarsertib on SW480 wild-type and SW480 SALL2 KO cells were determined by crystal violet Assay. Five thousand cells were seeded on 96-well plates and incubated with increasing concentrations of Silmitarsertib (0–40 μM) for 24 h at 37 °C. Cells were stained with 0,01% (p/v) crystal violet [19] and fluorescence (680 nm) was quantified using LI-COR ODYSSEY CLX equipment.

### IncuCyte Real-time live-cell cell death assay

SW480 wild-type and SW480 *SALL2* KO cells were treated with Silmitasertib 25 μM or DMSO. The cytotoxicity was analyzed in real-time using INCUCYTE S3 Live-Cell Analysis System and 30 nM Sytox Green dye. Dead cells were counted automatically every 1 h for 24 h. Cell death percentage was quantified with a cell-by-cell module and normalized by confluence using IncuCyte v2019B software from the Center for Advanced Microscopy (CMA Biobio), Universidad de Concepcion.

### Immunocytochemistry

HEK293 or U2OS cells were grown on coverslips coated with 0.01% poly-lysine. U2OS cells were transfected with pCMV2(NH)/SALL2-Flag, pCMV(NH)/ΔNSALL2-Flag or pCMV2(NH)/SALL2 mutant -Flag (SALL2 S763A; T778A; S802A or S806A-Flag). In the case of HEK293, cells were treated with TBB (50 µM) or DMSO for 7 h. Samples were fixed with 4% PFA and permeabilized with 0.1% Triton X-100. Cells were blocked with 3% BSA and incubated with the primary antibodies (anti-SALL2, anti-Flag, anti-CK2α/α’) in a humid dark chamber overnight. Anti-mouse and/or rabbit IgG conjugated to Alexa 488 or Alexa 555 secondary antibodies were used. Cell nuclei were stained with Hoechst 33342. Finally, coverslips were mounted with DAKO fluorescence mounting medium (Dako, Cat. No. # S3023) and visualized with a spectral confocal microscope (LSM780 NLO; Carl Zeiss) at CMA Bio Bio, Universidad de Concepcion. All analyses were performed in ImageJ and Zenlite (Carl Zeiss) software. For the morphological analysis, SW480 *SALL2* WT and SW480 *SALL2* KO cells were incubated with 25 μM Silmitarsertib for 24 h, and stained with Phalloidin-iFluor™ 488 Conjugate and DAPI. Photographs were obtained by confocal microscopy (Olympus IX81, Japan).

### Methuosis-like vacuoles analyses

SW480 *SALL2* WT and *SALL2* knockout cells were treated with 25 µM Silmitarsertib, and light images were obtained using the INCUCYTE S3 system (20× and 40× objectives). At least 100 cells from three or more images per condition were used for analyses. Cells were counted, and vesicle size was measured using “multi-point” and “lines” tools from ImageJ software. The number and size of vesicles per cell were analyzed from 40x images. To calculate the percentage of cells with vesicles, 20x images were analyzed. Finally, obtained data were analyzed with Prism GraphPad software.

### Organoids generation and culture

The colon from a B6- *Tg(Vil1-Cre*^*ERT2*^*)23Syr/J; Apc*^*tm1Tno*^ *Kras*^*tm4Ty*^*/J; Trp53*^*tm1Brn*^ (AKP) mouse (male, 25 weeks at day of sampling) was isolated and colon organoids were generated. These organoids were treated with 4-OHT to induce Cre activity. Recombination of conditional alleles was validated by PCR, and cells were cultured in media lacking R-spondin and EGF [76, 77]. Intestinal tumors were enzymatically dissociated to obtain single cells seeded in Cultrex Reduced Growth Factor Basement Membrane Extract (BME; R&D Systems, 3433-005-01) to generate organoids. These organoids were routinely cultured and passaged using BME. Domes were rinsed with 4 °C cold PBS to detach the BME. Samples were centrifuged at 1500 rpm for 5 min. The supernatant was aspirated, leaving a BME pellet and organoids. To break up the organoids, 150 µL of 4 °C cold PBS was added and pipetted vigorously and repeatedly. Subsequently, 5 mL of PBS was added and centrifuged at 1300 rpm for 3 min. The supernatant was aspirated, and organoids were suspended in BME (20 µL per dome) and plated on pre-warmed plates. Organoids were split at a ratio of 1:2 or 1:3 and maintained in advanced DMEM/F12 (Gibco, 12634028) with 1% penicillin–streptomycin (Sigma-Aldrich, P4458-100ML), 1% D-Glutamine (Gibco, 25030024), 1% HEPES (Sigma-Aldrich, h0887-100), 1× B27 (Gibco, 12587010), and 1× N2 (Gibco, 17502048). The experiment was conducted under German law with Bioethical approval via local authorities under protocol: DKFZ392.

### Generation of CRISPR-Cas9-mediated *Sall2* knockout tumor-derived organoids

Murine AKP organoids were cultured and broken up as mentioned above. On the day of electroporation, they were dissociated into single cells using 1× TrypLE Express (Invitrogen, Waltham, MA, USA, 12605010). Subsequently, 5 mL of PBS and 50 μL of FBS (Gibco, 16000044) were added and centrifuged at 1300 rpm for 3 min. The supernatant was aspirated, and organoids were resuspended in 100 μL OptiMEM (Gibco, 11520386). Ten µgs of pSpCas9(BB)-2A-GFP (PX458) (sgSall2 Fwd: CACCGGTGAGCGAGGAATTCGGTC, SgSall2 Rev: AAACGACCGAATTCCTCGCTCACC) were added, homogenized, and transferred to an electroporation cuvette. The NEPA21 electroporation system was used with the following settings: poring pulse: voltage 175 V, pulse length 5 ms, pulse interval 50 ms, number of pulses 2, decay rate 10%, polarity +. Transfer pulse: voltage 20 V, pulse length 50 ms, pulse interval 50 ms, number of pulses 5, decay rate 40%, polarity +/-. After electroporation, cells were recovered in 1 mL of cultured media supplemented with 10 μL of Y-27632 and incubated at room temperature for 30 min. Finally, cells were centrifuged at 1000 rpm for 3 min. The supernatant was removed, and the cell pellet was suspended in BME and plated on pre-warmed plates. Organoids were cultured for three days to select labeled organoids expressing the GFP protein by Fluorescent Activated Cell Sorting (FACS). Organoids embedded in BME were dissociated into single cells with 1× TrypLE Express for 5 min at room temperature. Single cells were resuspended in PBS with 1% FBS and passed through a 40 µm strainer. GFP positive cells were enriched using a BD FACSAria cell sorting system. After centrifugation (1300 rpm for 5 min at room temperature), cells were resuspended in BME and plated. Single organoids were picked with a 10 μL tip to select colonies and cultured alone in a 24-well plate until they reached optimal confluency to determine the knockout status by Sanger Sequencing.

### Cytotoxic assay and Silmitarsertib IC50 determination in tumor-derived organoid

Murine AKP organoids were seeded in a density of 3000 cells/well in a 24-well plate. After three days, culture media was removed and media supplemented with different concentrations of Silmitarsertib (0, 6.25, 12.5, 25, 50, and 100 μM). After 24 h, organoid images were taken using the CytoSmart Omni Imaging System (Axion BioSystems). The culture media was removed to measure the organoid’s viability, and 300 mL of Cell Titer Blue reagent mixed with advanced DMEM/F12 (ratio 1:5) was added to each well. The plate was incubated in culture conditions for 2 h, and fluorescence was measured at 560/590 nm.

### Statistical analyses

The data analysis was carried out in the GraphPad Prism 5 Program by means of the Welch test (*t*-test with Welch’s correction) unpaired to compare normal samples with different variances. To compare more than two groups, the data were analyzed using one-way analysis of variance (ANOVA) for samples that differ by only one factor (a single independent variable, e.g., treatment) or two-way for samples that differ by two factors. (e.g. concentration of a drug and treatment time). For graph of methuosis-like vesicules analysis, data were subjected to one-way ANOVA, using the Holm-Sidak’s multiple comparisons test correction. Comparisons were made in units relative to the control condition. *P*-values below 0.05 were considered significant. All the analysis was performed in Prisma GraphPad.

### Supplementary information

Supplemental material

SW480WT DMSO

SW480WT Silmitasertib

SW480SALL2KO DMSO

SW480SALL2KO Silmitasertib

Original Data File

checklist

report cell line validation 1

report cell line validation 2

## Data Availability

All datasets generated for this study can be found in the article/Supplementary material; further inquiries can be directed to the corresponding author. The full-length uncropped original western blots used in the manuscript are uploaded as a single ‘Supplemental Material’ file. Mass Spectrometry data at: https://zenodo.org/records/10475931.
