# Supplementary material for: Casein kinase 2 phosphorylates and induces the SALL2 tumor suppressor degradation in colon cancer cells
Source: Cell Death Dis. 2024 Mar 16;15(3):223. doi: 10.1038/s41419-024-06591-z (PMC10944491; doi:10.1038/s41419-024-06591-z)
Supplement: Supplementary file 1 — Supplemental material [file 41419_2024_6591_MOESM1_ESM.pdf]

Modified Residue	Condition	Total spectra	Modified spectra	% Modified peptides
T467	1: λ-Phosphatase	128	0	0
	2: *in vitro* no enzyme (basal)	116	0	0
	3: *in vitro* CK2α	124	0	0
	4: DMSO	208	4	**1,9**
	5: CX-4945	156	10	**6,4**
S763	1: λ-Phosphatase	74	0	0
	2: *in vitro* no enzyme (basal)	27	0	0
	3: *in vitro* CK2α	44	1	2,3
	4: DMSO	20	20	**100**
	# 5: CX-4945	81	0	0
T778	1: λ-Phosphatase	30	0	0
	2: *in vitro* no enzyme (basal)	3	0	0
	3: *in vitro* CK2α	32	0	0
	4: DMSO	19	8	**42,1**
	5: CX-4945	44	0	0
S802	1: λ-Phosphatase	333	0	0
	2: *in vitro* no enzyme (basal)	350	16	**4,6**
	3: *in vitro* CK2α	430	49	**11,4**
	4: DMSO	456	182	**39,9**
	5: CX-4945	594	107	**18**
S806	1: λ-Phosphatase	333	0	0
	2: *in vitro* no enzyme (basal)	350	33	**9,4**
	3: *in vitro* CK2α	430	71	**16,5**
	4: DMSO	456	206	**45,2**
	5: CX-4945	594	75	**12,6**

Supplementary Table 1

Supplementary Table 2: Primers used in site-directed mutagenesis.

Name	Sequence	Tm (ºC)	Substitution (DNA)	Mutation (protein)
S763 For	5’-GAAGAGGAGTTGGCTGAGG AGGAGG-3'	75.1	c.T2329>G (TCT>GCT)	p.S763A
S763 Rev	5'-CCTCCTCCTCAGCCAACTC CTCTTC-3'			
T778 For	5’-GAAGAGGAAGATGTGGCTG ATGAAGATTCC-3'	74.8	c.A2374>G (ACT>GCT)	p.T778A
T778 Rev	5'-GGAATCTTCATCAGCCACAT CTTCCTCTTC-3'			
S802 For	5’-CAGTGAGAGGTGATGCAGA AGAGGCATC-3'	75.8	c.T2446>G (TCA>GCA)	p.S802A
S802 Rev	5'-GATGCCTCTTCTGCATCACC TCTCACTG-3'			
S806 For	5'-GATTCAGAAGAGGCAGCTG GGGCAGAGGAG-3'	80.3	c.T2458>G (TCT>GCT)	p.S806A
S806 Rev	5' -CTCCTCTGCCCCAGCTGCC TCTTCTGAATC-3'			
S802A (B) For	5' -CTCCTCTGCCCCAGCTGCC TCTTCTGAATC-3'	77.2	c.T2446>G (TCA>GCA)	p.S802A- sobre mutación S806A
S802A (B) Rev	5'- CAGTGAGAGGTGATGCAGA AGAGGCAGC-3'			
A763D For	5'-GAAGAGGAGTTGGATGAG GAGGAGGAAG-3'	75.8	c.C2330>A (GCT>GAT)	p.A763D
A763D Rev	5'-CTTCCTCCTCCTCATCCAA CTCCTCTTC-3'			
A778D For	5'-GAAGAGGAAGATGTGGATGATGAAGATTCCCTG-3'	76.7	c.C2375>A (GCT>GAT)	p.A778D
A778D Rev	5'-CAGGGAATCTTCATCATCCACATCTTCCTCTTC-3'			
A802D For	5'-CAGTGAGAGGTGATGATGAAGAGGCAGCTGG-3'	79.0	c.CA2456>A T (GCA>GAT)	p.A802D
A802D Rev	5'-CCAGCTGCCTCTTCATCATCACCTCTCACTG-3			
A806D For	5'-GATGAAGAGGCAGATGGGGCAGAGGAG-3'	77.1	c.C2459>A (GCT>GAT)	p.A802D sobre mutación A802D
A806 D Rev	5'-CTCCTCTGCCCCATCTGCCTCTTCATC-3'			

Supplementary Figure 1

A

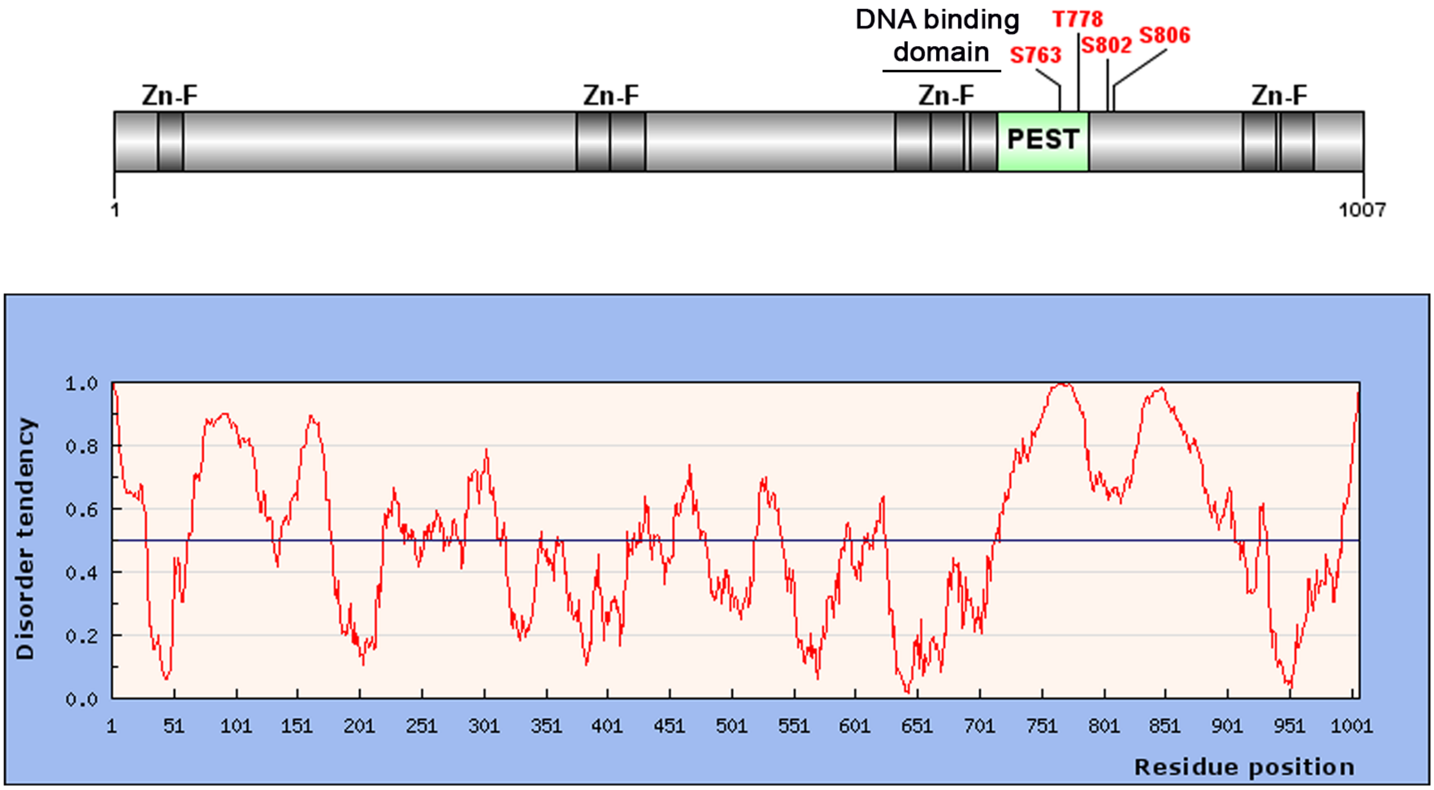

B

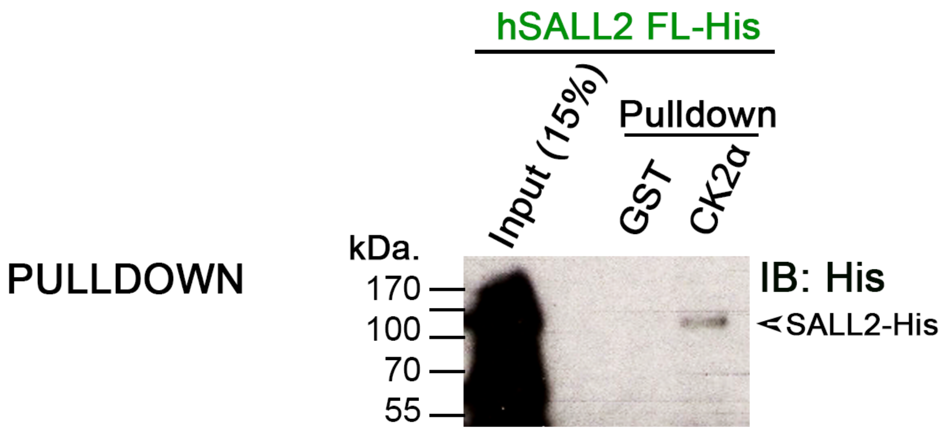

Supplementary Figure 2

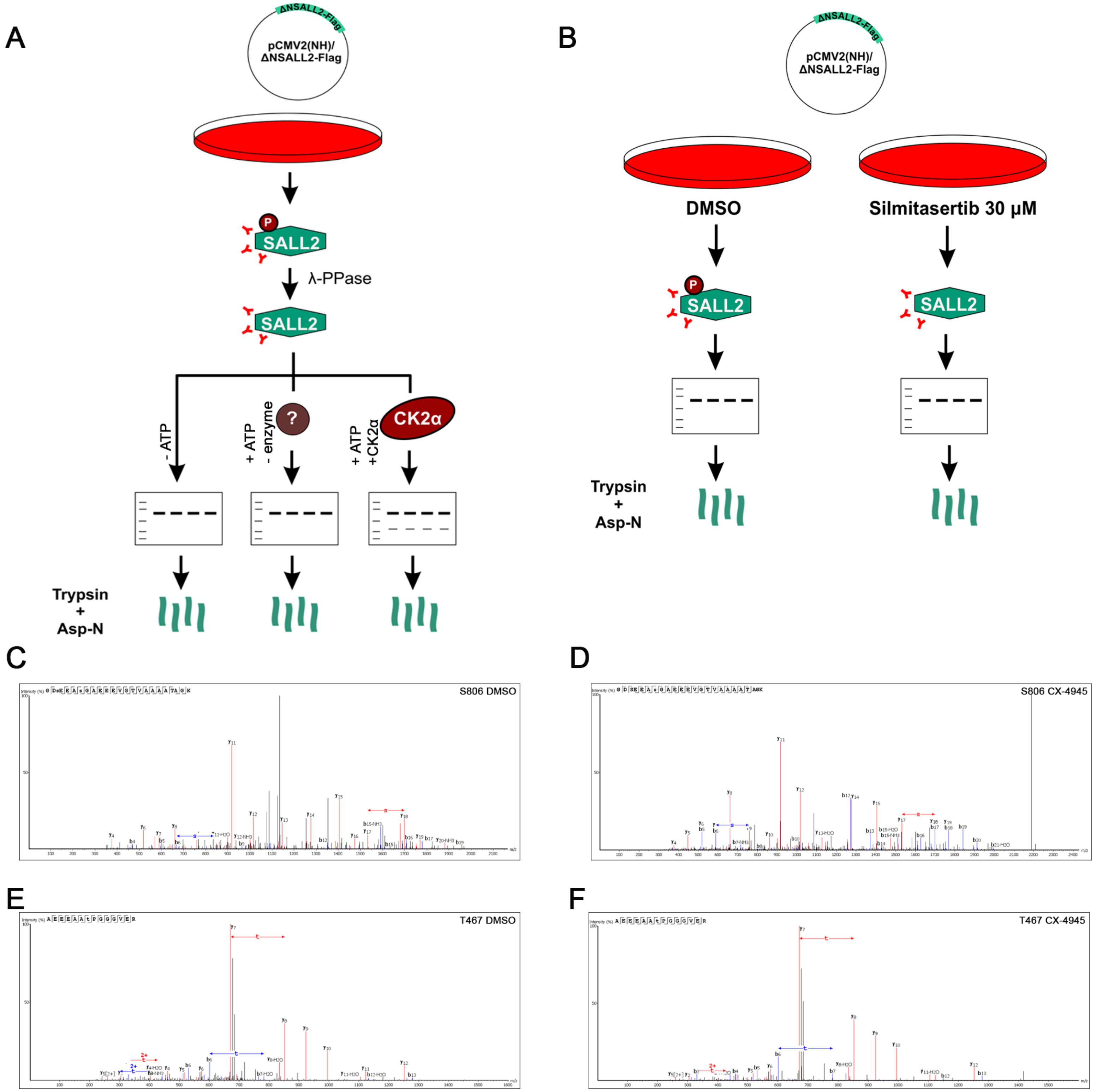

Supplementary Figure 3

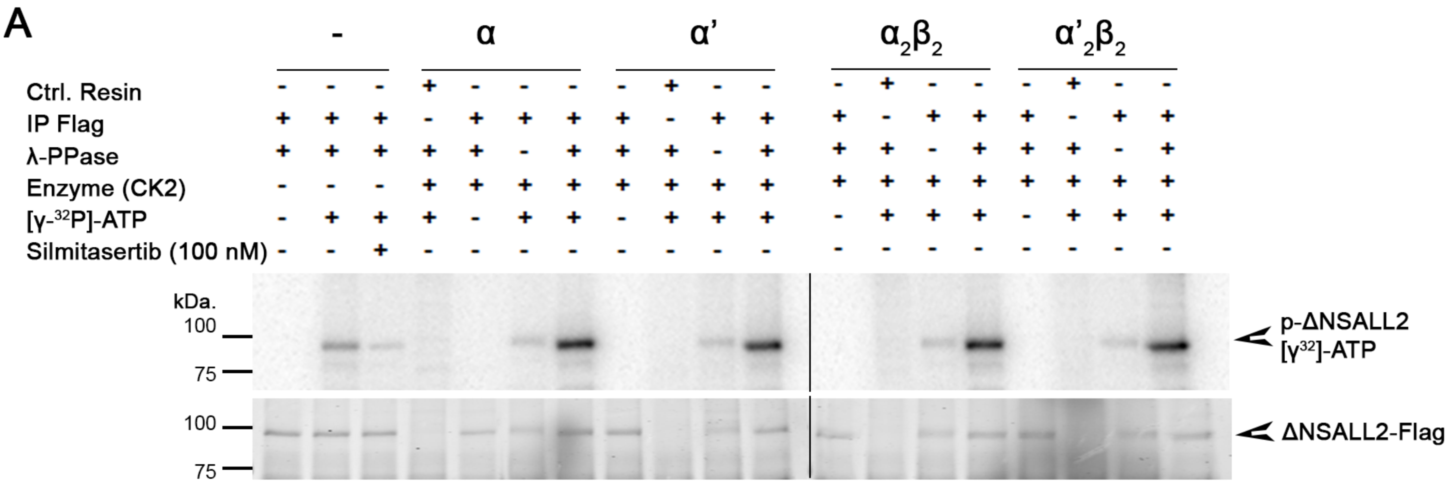

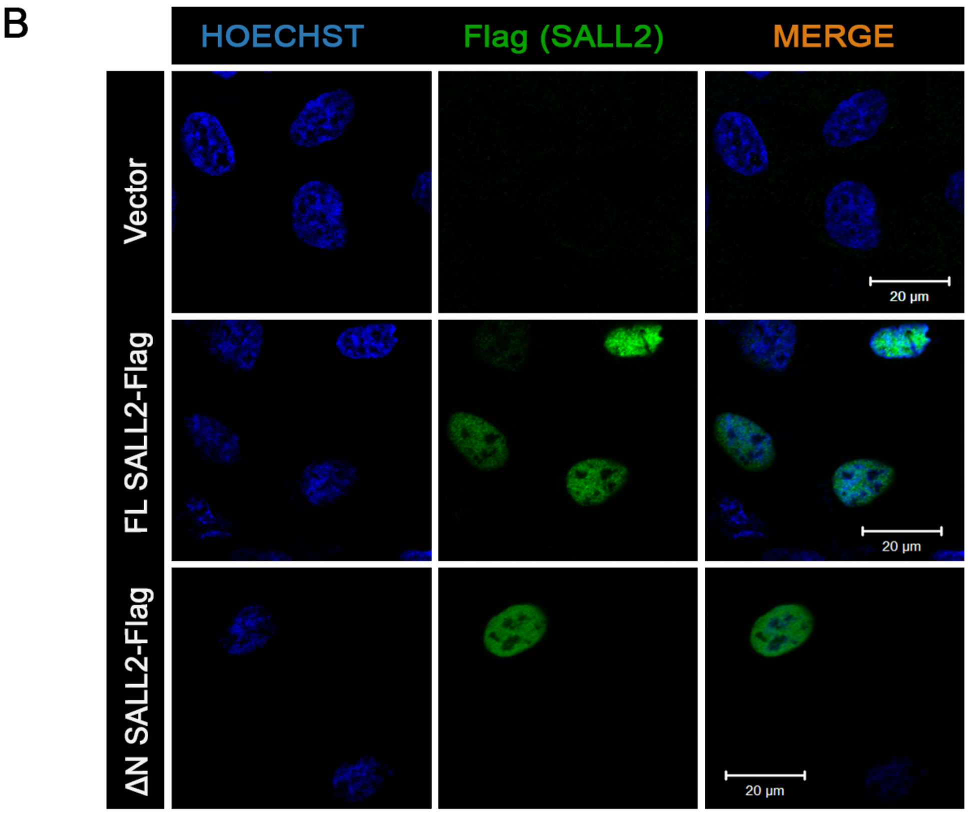

Supplementary Figure 4

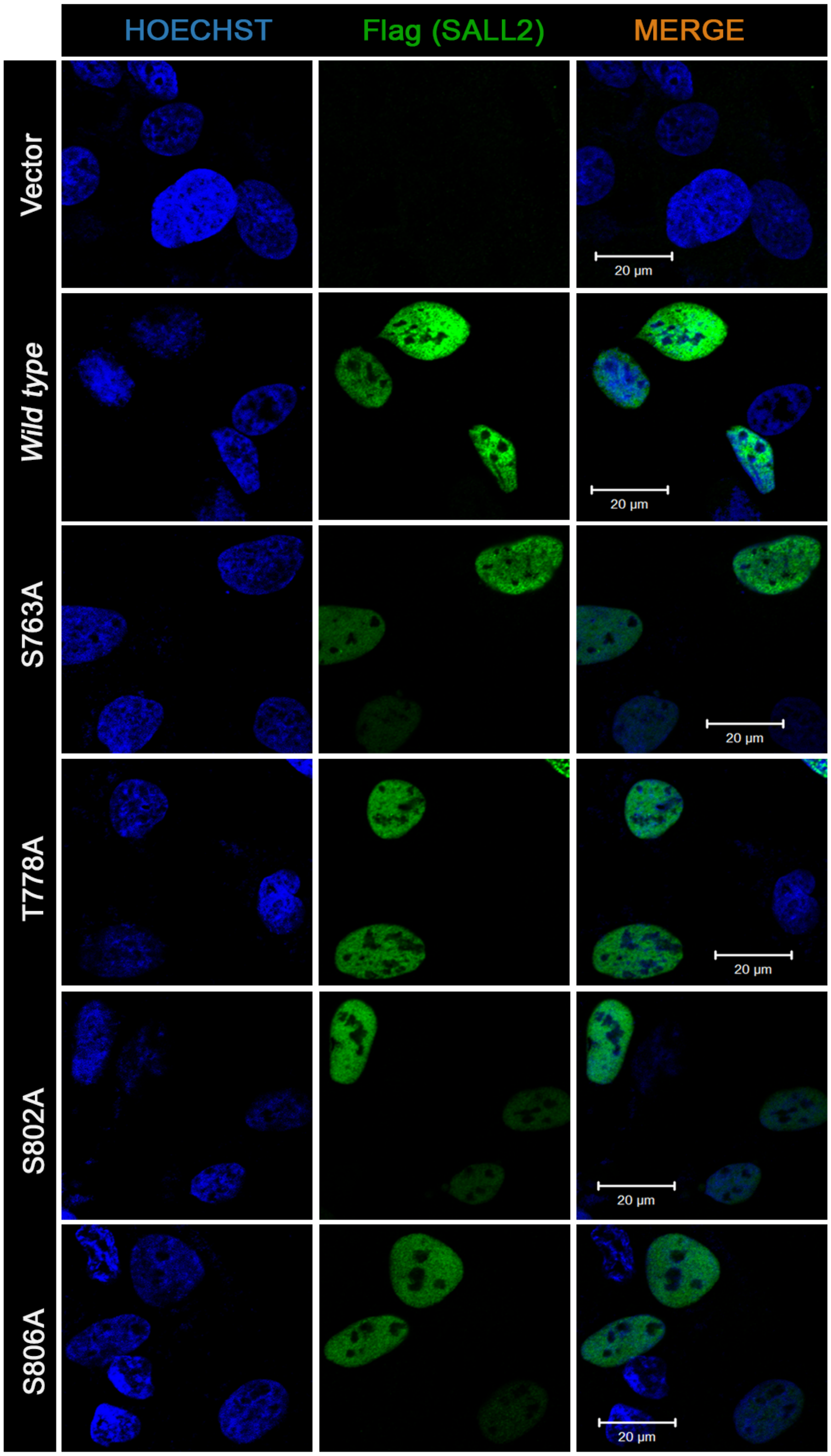

Supplementary Figure 5

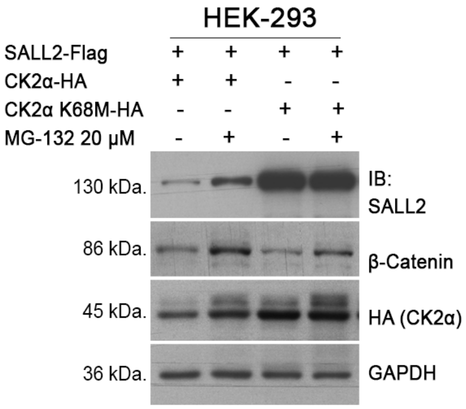

Supplementary Figure 6

A

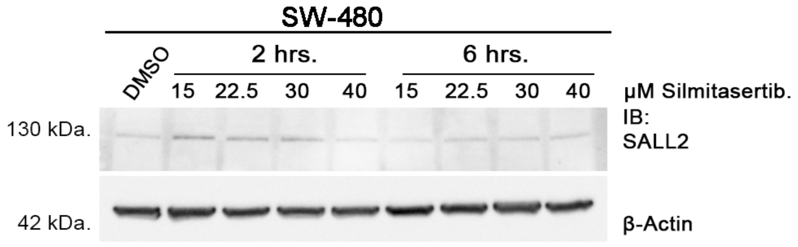

B

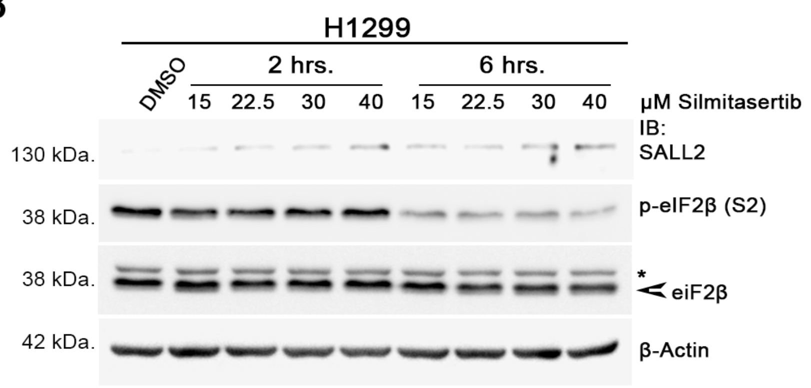

C

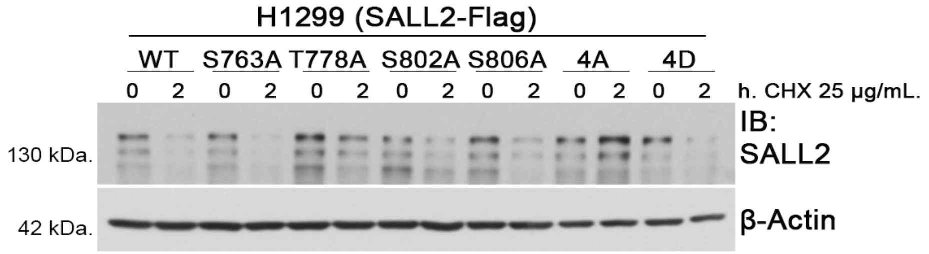

D

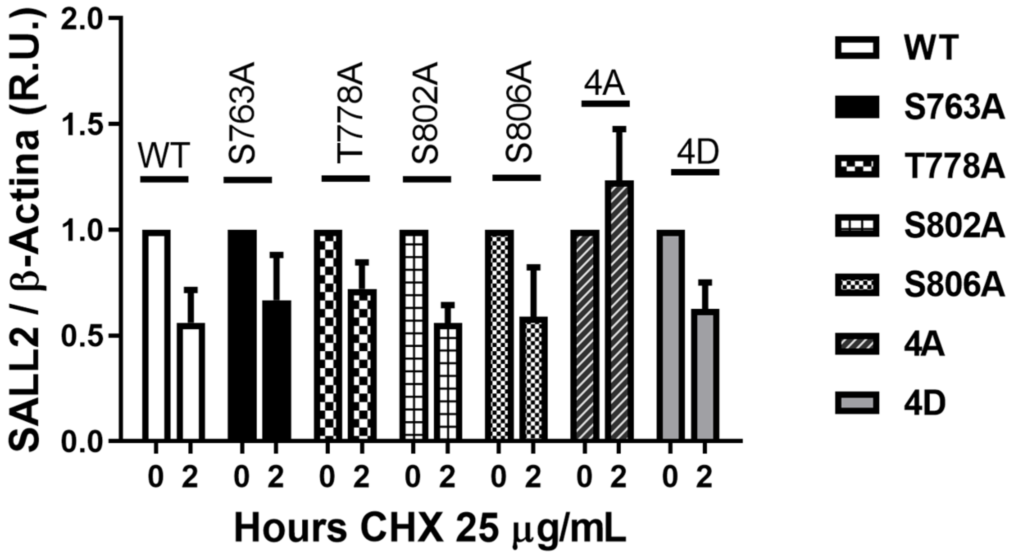

Supplementary Figure 7

A

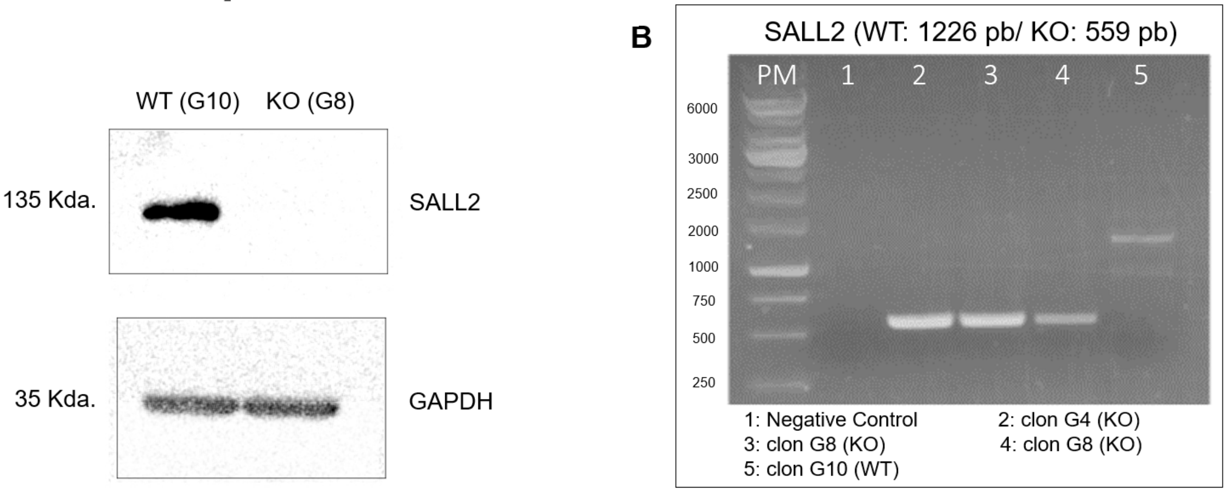

C

CLUSTAL format aligment by MAFFT FFT-NS-i (v7.471)

```
Intron IA-2       CCCACCCCCAACTAGCGGTTACTGGCCTCCTTGTTAATTAGAGCTCGGTATACCCAACC
SW480 G10 (WT)    CCCACCCCCAACTAGCGGTTACTGGCCTCCTTGTTAATTAGAGCTCGGTATACCCAACC
SW480 G8 (KO)     CCCACCCCCAACTAGCGGT----------------------------------------

Exon 2            CTGTTGCTTGGCTCCTTAGGCCAGACGGTGGGTGCCCCTGCAGTCCCTACAGAGCTACCT
SW480 G10 (WT)    CTGTTGCTTGGCTCCTTAGGCCAGACGGTGGGTGCCCCTGCAGTCCCTACAGAGCTACCT
SW480 G8 (KO)    -----------------------------GGTGGGTGCCCCTGCAGTCCCTACAGAGCTACCT
```

Supplementary Figure 8

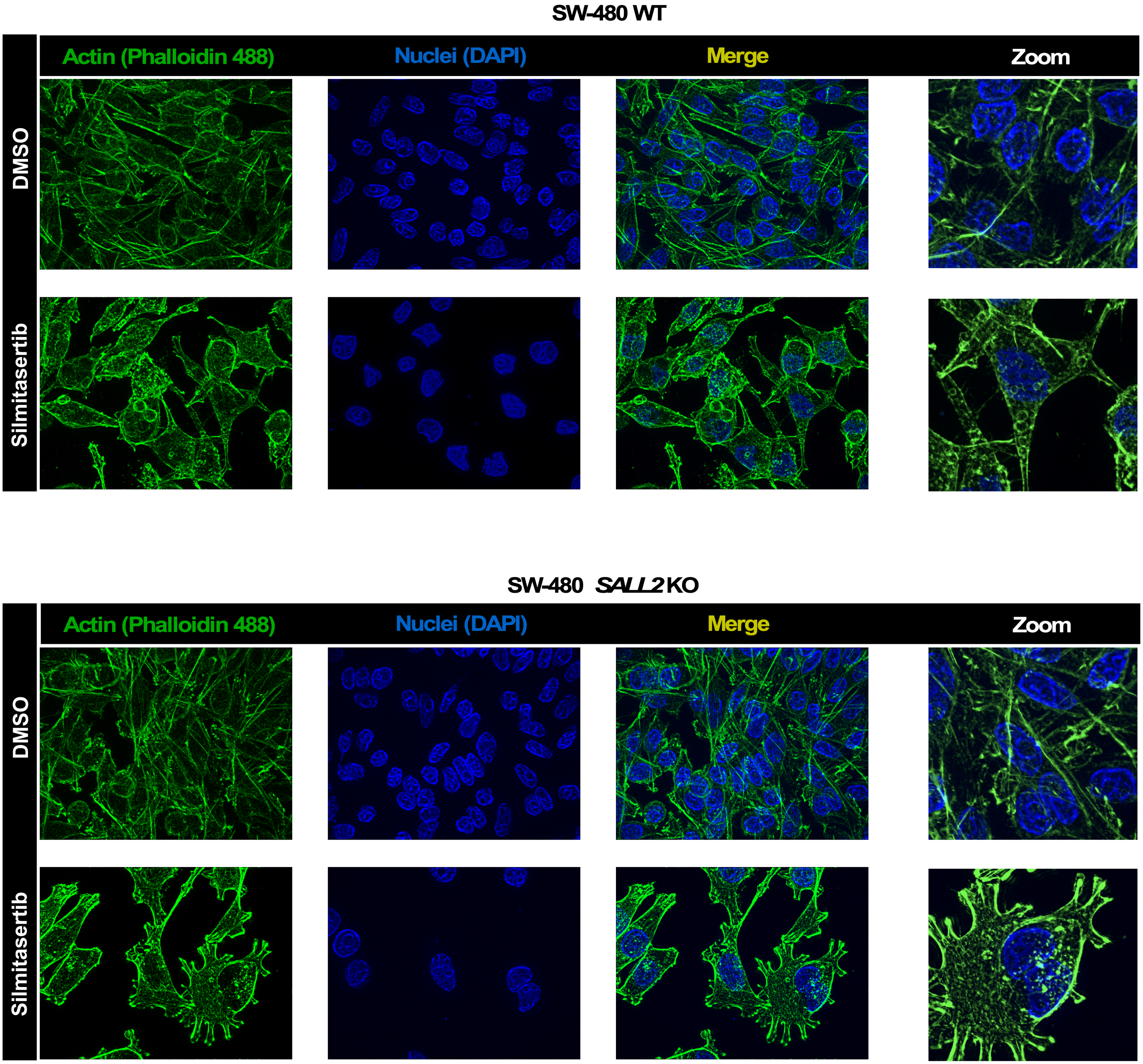

Supplementary Figure 9

A

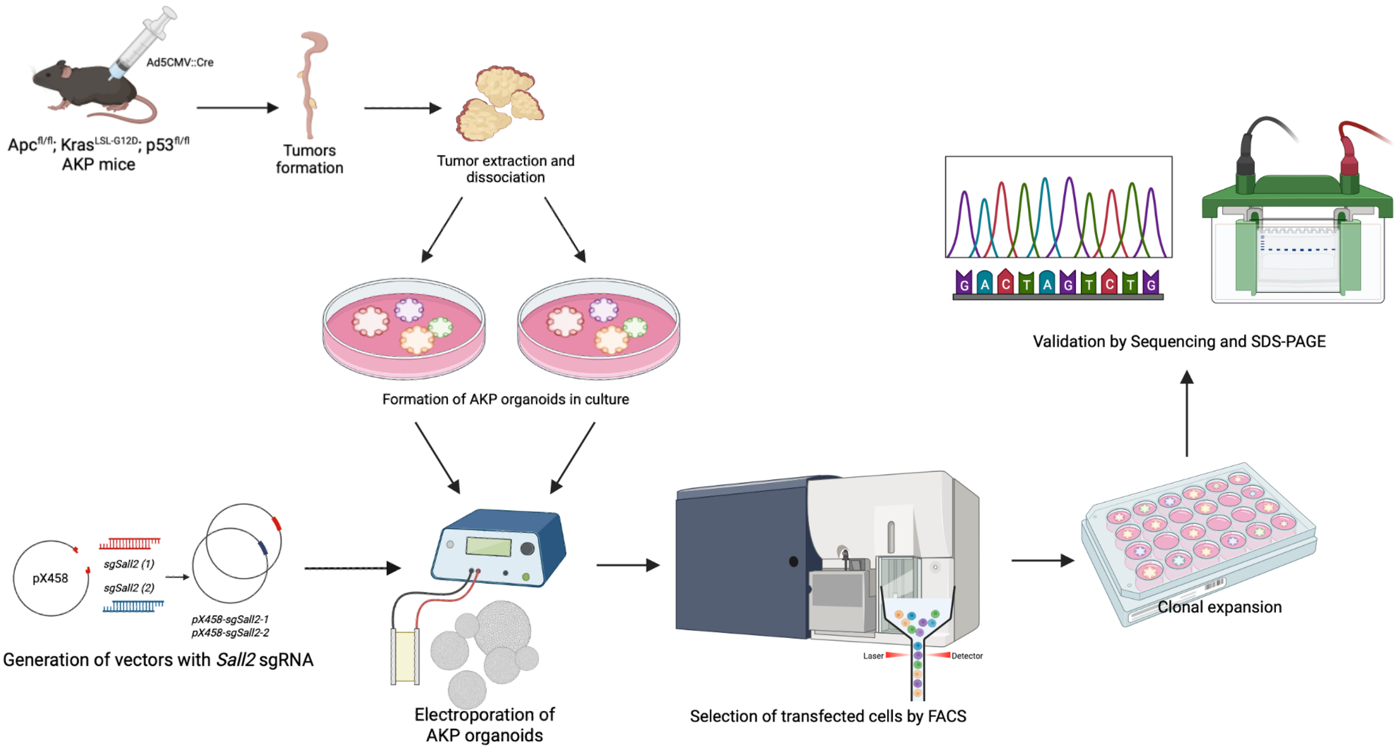

B

CLONE 5 – sgSall2

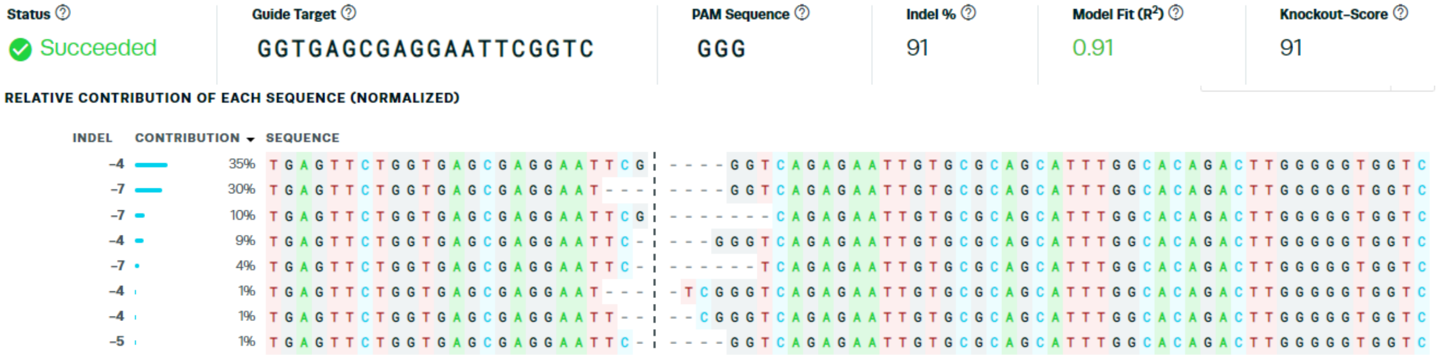

C

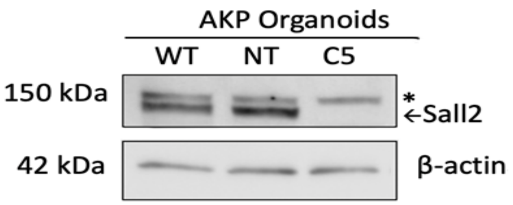

Supplementary Figure 10

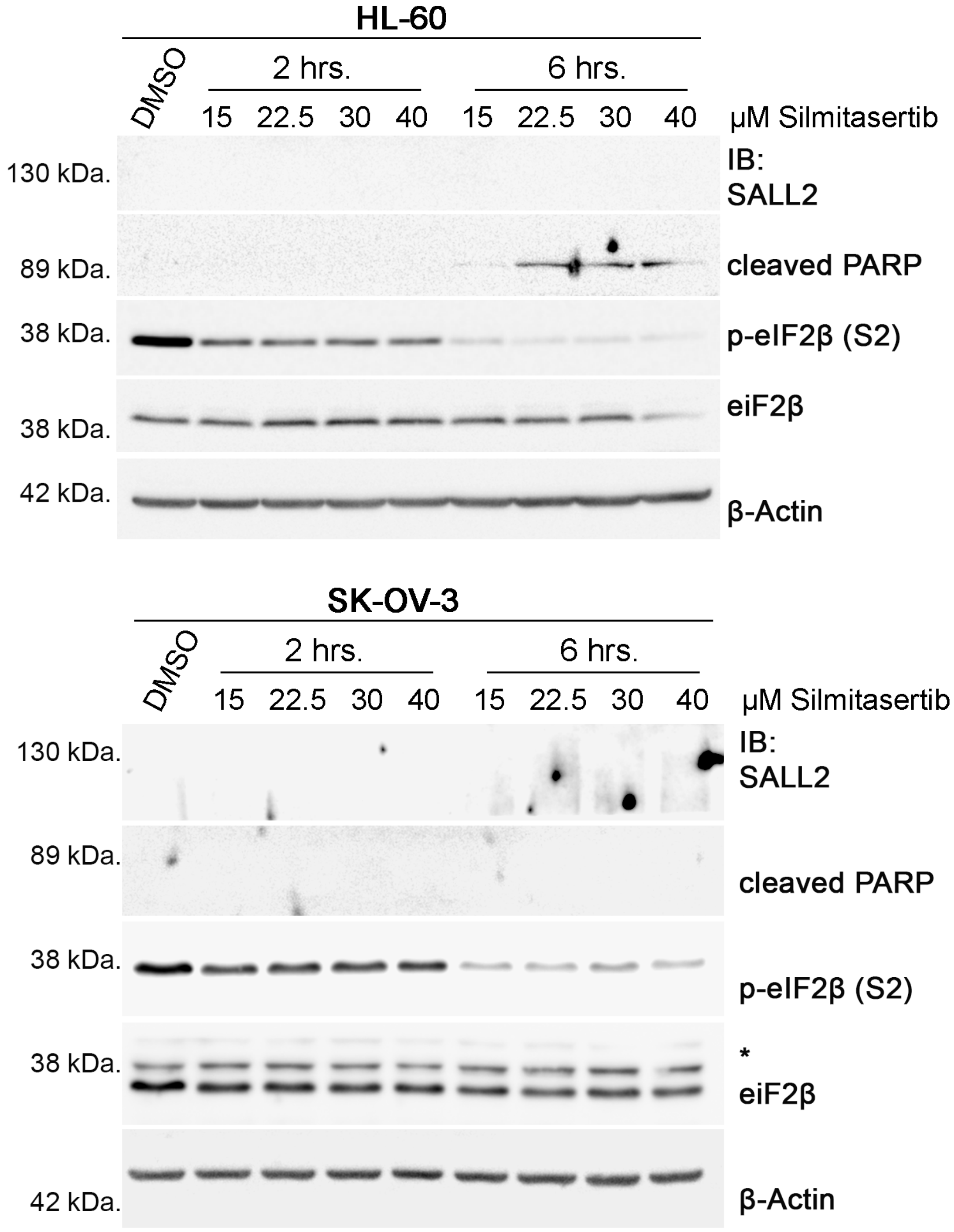

Legends

Supplementary Table 1. Mass Spectrometry Data. Those sites detected in ionic fragments whose intensity exceeds 5% relative abundance are highlighted in bold. CK2 sites are highlighted in red. The T467 site highlighted does not correspond to a CK2-mediated phosphorylation site.

Supplementary Table 2. Sequences from Forward (For) and Reverse (Rev) primers used in site-directed mutagenesis.

Supplementary Fig. 1. A. SALL2 protein disorder prediction with the IUPred tool. The Y axis represents the score delivered by the program, and the X axis shows the sequence length. Scores over 0.5 predict disorder. The regions with a lower degree of disorder correspond to the location of the second and third pair of Zinc finger motifs. **B. GST-Pulldown assays** using the human GST-CK2α protein as "bait" and the hSALL2 FL-His as "prey." As a control, the resin was used only with GST. Protein interaction was assessed by western blot. Representative figure from two independent experiments.

Supplementary Fig. 2. A and B. Workflow for mass spectrometry. We overexpressed and immunoprecipitated ΔN SALL2-Flag to identify SALL2 residues phosphorylated by CK2α *in vitro* (**A**) and in a CK2-dependent manner in cells (**B**). Next, we performed sequential *in-gel* digestion with trypsin and Asp-N proteases, as suggested by the MS-Digest tool from the protein Prospector program. **C-F. Mass spectrum of peptides** with phosphorylation at S806 and T467 treated with DMSO (**C, E**) and Silmitarsertib (CX-4945) (**D, F**) from ΔNSALL2-Flag protein immunoprecipitated from cells.

Supplementary Fig. 3. A. *In vitro* phosphorylation of ΔNSALL2-Flag by recombinant CK2. The ΔNSALL2-Flag protein was immunoprecipitated from Flp-InTM T-RExTM U2OS cells with an anti-Flag antibody. The protein was dephosphorylated with λ-Phosphatase and incubated with [γ32]-ATP in the absence of enzyme (-), in the absence of enzyme and with Silmitarsertib, or in the presence of recombinant CK2α, CK2α', CK2α2β2, or CK2α'2β2. As negative controls, reactions without [γ32]-ATP or without substrate were carried out. The arrows indicate the bands corresponding to ΔNSALL2-Flag in the autoradiography (upper part) and the colloidal Coomassie stain (lower part). **B. Subcellular localization of SALL2-Flag and ΔNSALL2-Flag.** U2OS cells were transfected in a 100 mm dish with plasmid DNA, corresponding to empty vector (Vector), pCMV2(NH)/Sall2-Flag (FL) or pCMV2(NH)/ΔNSall2-Flag (ΔN). Cells were analyzed by immunofluorescence with an anti-Flag antibody. In blue is the nucleus (Hoechst). In green are the SALL2-Flag FL, and ΔNSALL2-Flag, proteins. Merge: overlapping of individual channels. Size bar: 20 μm. Representative figure from three independent experiments.

Supplementary Fig. 4. Mutations in the SALL2 phosphoacceptor sites do not alter the protein's nuclear localization. U2OS cells were transfected with pCMV2(NH)-Flag as empty vector (Vector) or with plasmids pCMV2(NH)/Sall2-Flag encoding SALL2-Flag wild type or individually mutated phosphoacceptor residues to alanine: S763A, T778A, S802A or S806A. Cells were fixed with paraformaldehyde 40 hours after transfection, and Immunofluorescence detected SALL2 with an anti-Flag antibody (green). In blue is the

nucleus (Hoechst). Merge overlaps of individual channels. Size bar: 20 μm. Representative figure from three independent experiments.

Supplementary Fig. 5. CK2 activity promotes the proteasomal degradation of SALL2 in HEK 293 cells. HEK 293 cells were transfected with wild-type (CK2α-HA) and mutant (CK2α K68M-HA) CK2 plasmids and treated with/without 20 μM MG-132. Endogenous SALL2 protein levels were assessed by western blot. β catenin, a CK2 substrate whose stability is positively regulated by CK2 phosphorylation, was used as a positive control. Representative figure from three independent experiments.

Supplementary Fig. 6. Pharmacological inhibition of CK2 restores SALL2 in SW480 (**A**) and H1299 (**B**) cancer cell lines. Cells were treated with different concentrations of the CK2 inhibitor, Silmitarsertib, for 2 and 6 hours. SALL2 levels and CK2 activity markers were assessed by western blot. The arrow in eIF2β shows the band corresponding to the total protein. Representative figure from three independent experiments. **C**. **SALL2 non-phosphorylatable mutant presents greater stability than wild-type counterpart**. H1299 cells were transfected with constructs encoding SALL2-Flag wild type (WT), S763A, T778A, S802A, S806A, S763A/T778A/S802A/S806A (4A, non-phosphorylatable mutant) or SALL2-Flag S763D/T778D /S802D/S806D (4D, phosphomimetic mutant). Cells were treated with 25 μg/mL cycloheximide (CHX) for 0 and 2 hours, and SALL2-Flag was immunodetected by western blot. **D.** Quantification of SALL2-Flag in relation to β-actin levels. N=3

Supplementary Fig. 7. Validation of *SALL2* KO CRISPR/Cas9clones. **A.** Western Blot from lysates of SW480 control and *SALL2* KO CRISP/Cas9 clones. GAPDH is the loading control. **B.** Amplicons obtained from gDNA samples. The 1226 bp fragment corresponds to the undeleted *SALL2* locus, and the 559 bp fragment corresponds to the product of the deleted *SALL2* locus. A sample without DNA was used as a negative control. **C.-** Sequence analysis of SW480 genomic DNA samples. A segment of Intron 1A-2 sequences is compared between *SALL2* control and *SALL2* KO clone G8. The 20-nucleotide target sequence for the Cas9/sgRNA complex is in yellow, and the PAM site is in a red square. The top sequence of each set corresponds to the *SALL2* genomic sequence (ENSG00000165821).

Supplementary Fig. 8. Silmitarsertib induces a SALL2-dependent methuosis-like phenotype in SW480 cancer cells. The SW480 SALL2 wild-type (clone G10) and SALL2 knockout (clone G8) were treated with 25 μM Silmitarsertib for 24 hours. The methuosis-like phenotype was analyzed by immunocytochemistry using Phalloidin-iFluor™ 488 for actin cytoskeleton and DAPI for nuclei. Microphotographs were obtained by confocal microscopy (Olympus IX81, Japan) using 60X objective.

Supplementary Fig. 9. Validation of AKP-Sall2KO organoids. A. Genetically engineered B6- Tg(*Vil1-Cre*^{ERT2})23Syr/J; *Apc*^{tm1Tno} *Kras*^{tm4Ty}/J; *Trp53*^{tm1Brn} (AKP) mice were treated with 4-OHT to induce Cre activity and tumor formation. Intestinal tumors were enzymatically dissociated to obtain single cells. AKP organoids formed by culturing single cells in BME. Organoids were electroporated with vectors carrying Sall2 sgRNAs, sorted by

FACS, and single cells were seeded for clonal expansion. **B**. The knockout status (AKP Sall2 KO) was validated by Sanger sequencing and the Inference of CRISPR Edits (ICE) tool from Synthego. Sequences of Sall2 KO clones (shown clone 5) and wild-type AKP were uploaded and compared using the knockout score and the relative contribution of each sequence. **C**. Western blot from AKP Sall2 KO organoid clone 5 (C5), the non-targeting control (NT), and the wild-type AKP organoid (WT). Asterisk (*) is used to label nonspecific bands in the western blot.

Supplementary Fig. 10. Silmitasertib does not increase SALL2 in HL-60 and SKOV3 cancer cell lines. HL-60 and SKOV3 cells were subjected to CK2 inhibition for 2 and 6 hours with various concentrations of Silmitarsertib. SALL2 protein levels were assessed by western blot. Phosphorylation of eIF2β (p- eIF2β) was used as a positive control of CK2 inhibition and β-Actin as loading control. Representative figure from three independent experiments.
