# Supplementary material for: Casein kinase 2 phosphorylates and induces the SALL2 tumor suppressor degradation in colon cancer cells
Source: Cell Death Dis. 2024 Mar 16;15(3):223. doi: 10.1038/s41419-024-06591-z (PMC10944491; doi:10.1038/s41419-024-06591-z)
Supplement: Supplementary file 9 — report cell line validation 2 [file 41419_2024_6591_MOESM9_ESM.pdf]

genYtec
en el ADN de la genética

November 30, 2023
Santiago, Chile

Genetic Profile Validation by STR analysis:

Sample type: DNA extracts from cell lines

Samples description:
The sample was received at Genética y Tecnología SpA. On November 22, 2023 and was labeled:
LC3 (Informed concentration: 151.5 ng/ul)

The technical process to carry out this comparative study of DNA polymorphisms can be performed by different methods. One of them employs the Polymerase Chain Reaction Amplification (PCR). Using this technology, specific points of DNA are amplified millions of times in an in-vitro reaction, obtaining very small pieces of DNA that are then separated by size and "measured" for comparison.

In the present study, molecular genetic analysis of the STR loci **D3S1358, D13S317, Penta E, D16S539, D18S51, D2S1338, CSF1PO, Penta D, TH01, vWA, D21S11, D7S820, D5S818, TPOX, D8S1179, D19S433, FGA**, and the sex marker **Amelogenin** was performed, by AMP-FLP (Amplified Fragment Length Polymorphism) using the PowerPlex® Fusion System (Promega Corporation) and electrophoretic separation of the alleles in an automatic genetic analyzer model ABI 310, Applied Biosystems.

To complete the analysis, the profile obtained was compared with the reference profile, which according to background, belongs to the **SW480** cell line.

Results:

LOCUS	AMEL		D3S1358		D13S317		Penta E		D16S539		D18S51		D2S1338		CSF1PO	
LC3	X	X	15	15	12	12	10	10	13	13	13	13	17	24	13	14
SW480	X	X	15	15	12	12	10	10	13	13	13	13	17	24	13	14

LOCUS	Penta D		TH01		vWA		D21S11		D7S820		D5S818		TPOX		D8S1179	
LC3	9	15	8	8	16	16	30	30.2	8	8	13	13	11	11	13	13
SW480	9	15	8	8	16	16	30	30.2	8	8	13	13	11	11	13	13

LOCUS	D19S433		FGA	
LC3	13	13	24	24
SW480	13	13	24	24

Common alleles between the two profiles are indicated in bold.

GENETICA Y TECNOLOGIA S.P.A.
RUT:78.340.830-9
FONO: 26335269

BQ. M. Victoria Aguayo A.
Professional Responsible
Genética y Tecnología SpA.

Genética y Tecnología SpA. - Coronel Santiago Bueras 160-B, Santiago Chile
Fono: +56 2 28893100 +56 9 71370018
www.genytec.cl genytec@genytec.cl
